# Quantifying the effect of uncertainty in input parameters in a simplified bidomain model of partial thickness ischaemia

**DOI:** 10.1007/s11517-017-1714-y

**Published:** 2017-09-20

**Authors:** Barbara M. Johnston, Sam Coveney, Eugene T. Y. Chang, Peter R. Johnston, Richard H. Clayton

**Affiliations:** 1 Queensland Micro- and Nanotechnology Centre and School of Natural Sciences, Griffith University, Nathan, QLD 4111 Australia; 2 Department of Physics and Astronomy, University of Sheffield, Sheffield, UK; 3 Department of Computer Science and INSIGNEO Institute for in-silico Medicine, University of Sheffield, Sheffield, UK

**Keywords:** Ischaemia, ST depression, Bidomain model, Conductivity values, Gaussian process emulators

## Abstract

Reduced blood flow in the coronary arteries can lead to damaged heart tissue (myocardial ischaemia). Although one method for detecting myocardial ischaemia involves changes in the ST segment of the electrocardiogram, the relationship between these changes and subendocardial ischaemia is not fully understood. In this study, we modelled ST-segment epicardial potentials in a slab model of cardiac ventricular tissue, with a central ischaemic region, using the bidomain model, which considers conduction longitudinal, transverse and normal to the cardiac fibres. We systematically quantified the effect of uncertainty on the input parameters, fibre rotation angle, ischaemic depth, blood conductivity and six bidomain conductivities, on outputs that characterise the epicardial potential distribution. We found that three typical types of epicardial potential distributions (one minimum over the central ischaemic region, a tripole of minima, and two minima flanking a central maximum) could all occur for a wide range of ischaemic depths. In addition, the positions of the minima were affected by both the fibre rotation angle and the ischaemic depth, but not by changes in the conductivity values. We also showed that the magnitude of ST depression is affected only by changes in the longitudinal and normal conductivities, but not by the transverse conductivities.

**Electronic supplementary material:**

The online version of this article (10.1007/s11517-017-1714-y) contains supplementary material, which is available to authorized users.

## Introduction

Myocardial ischaemia is a condition that results from reduced blood flow to the heart from the coronary arteries. Chest pain and symptoms of myocardial ischaemia are one of the most common reasons for patients to present to hospital emergency departments [32]. Since one method for detecting myocardial ischaemia is elevation or depression of the ST segment of the electrocardiogram (ECG), a comprehensive understanding of the biophysical basis of these changes is an important goal for researchers in this area.

When evaluating various anti-ischaemia interventions, ideally clinicians would be able to use ECG ST-segment changes to determine whether the presentation is acute full-thickness (transmural) ischaemia or whether the ischaemia is partial thickness (subendocardial). This distinction is important because transmural ischaemia corresponds to full occlusion of the coronary artery, and partial occlusion of the coronary artery is thought to be related to ST depression via subendocardial ischaemia [44]. From a clinical point of view, the importance of locating the ischaemic region is also clear because of the connection with arterial blockage.

It is well-accepted that transmural ischaemia results in epicardial ST elevation, which can also be detected on the body surface [31]. However, epicardial ST depression and its observation on the body surface are not as well-correlated with subendocardial ischaemia [44].

Various studies have found differences in the position of areas of ST depression and elevation. For example, some studies [30, 37] suggest that the maximum ST depression occurs over a lateral boundary between normal and ischaemic tissue in subendocardial ischaemia, with or without elevation directly over the ischaemic region. Others [21] agree with this basic finding but suggest that both the magnitude and location of ST depression are sensitive to changes in the conductivity values used, in particular their anisotropy.

In the last ten or so years, a large number of studies have used computer modelling to investigate aspects of the depression and elevation of the ST segment of the ECG; see, for example, [5, 6, 10, 22, 25, 39, 40, 43]. Some of these studies have examined the roles that tissue anisotropy and local fibre direction play in the injury currents that flow between healthy and ischaemic tissue, and have proposed mechanisms for the current flows that explain the formation of ST depression and elevation [5, 21, 22, 40].

The various input parameters to these models are not known with any great certainty and this is particularly so for the bidomain conductivity values for cardiac ventricular tissue. In the bidomain model, the tissue is represented as consisting of intracellular (*i*) or extracellular (*e*) spaces with conduction in three orthogonal directions. Since cardiac tissue consists of strands of cardiac fibres that make up ‘sheets’ that rotate relative to one another through the ventricular wall [20], these directions are defined as longitudinal (*l*) to/along the fibres, transverse (*t*) to/across the fibres within the sheet and normal (*n*)/perpendicular to the sheets, giving conductivities *g*_*pq*_,*p* = *i*,*e*,*q* = *l*,*t*,*n*.

Some sets of these cardiac conductivities have been experimentally determined and others are based on theoretical models, but, even if they were all experimentally determined, variations in experimental conditions, measurement accuracy, modelling assumptions and inter-subject differences would ensure that there would still be uncertainty in the parameters. Also, it is known that conductivity values change during the time course of ischaemia [40, 56], due to the collapse of interstitial space, cell swelling and the closure of gap junctions, and this justifies studying the effect of varying the input conductivities over quite a wide range.

One approach to studying the effect of uncertainty on input parameters is a population of models approach [41], in which a large number (say 10^5^) of model runs are used to explore the parameter space, and model calibration is done by discarding those runs that fall outside the range of observed outputs. Another is a Monte-Carlo approach, which also uses large numbers of model runs with different sets of input parameters. Still another approach, the one used in this study, is the construction of emulators (surrogate models). In this method, a much smaller number of model runs is used to construct a fast running emulator (a statistical model) of the original model (the simulator). The following are examples of emulators that have been used in the cardiac field: those constructed by generalised polynomial chaos [17, 26, 58], partial least squares [53] and Gaussian process emulators [12, 29].

The aim of the study was to deploy these uncertainty and sensitivity analysis techniques on a simplified model of subendocardial ischaemia that does not include the additional complexity of anatomically detailed representations of the heart and torso. We systematically examined the effect of uncertainty in various input parameters (fibre rotation angle, ischaemic depth, blood conductivity and the six bidomain conductivities) on the form of the epicardial potential distributions (EPDs) produced during the ST segment in a bidomain model of subendocardial ischaemia. We achieved this by examining the sensitivity of various outputs that characterise the EPD, such as maximum and minimum potentials and positions of the maximum and minimum, to changes in the input parameters.

Novel aspects of this study include the demonstration of the use of Gaussian process emulators in a tissue level model and the systematic examination of the effect of six, rather than four, conductivities on the outputs, as well as a study into the effect of uncertainty on fibre rotation angle and ischaemic depth on the position of the minimum potentials in the EPD.

## Methods

### Governing equations and model geometry

During the ST segment of the electrocardiogram, the passive bidomain equation [2, 61] can be used to model the electric potential in the extracellular space *ϕ*_*e*_, in a region of cardiac tissue, giving

$$\nabla \cdot \left( \textbf{M}_{i} + \textbf{M}_{e} \right) \nabla \phi_{e} = -\nabla \cdot \textbf{M}_{i} \nabla \phi_{m} $$

where *ϕ*_*m*_ is the transmembrane potential distribution and **M**_*p*_(*p* = *i*,*e*) are conductivity tensors in the intracellular and extracellular spaces, respectively. These tensors are of the form **M**_*p*_ = **AG**_*p*_ **A** ^*T*^(*p* = *i*,*e*), where **G**_*p*_ is a diagonal matrix with bidomain conductivity values (*g*_*pq*_,*q* = *l*,*t*,*n*) on the diagonal and **A** is a rotation matrix which maps the local fibre direction into the global coordinate system. We also assumed that the cardiac tissue is in contact with a blood mass in which the potential in the blood satisfies ∇^2^ *ϕ*_*b*_ = 0.

**Fig. 1:**
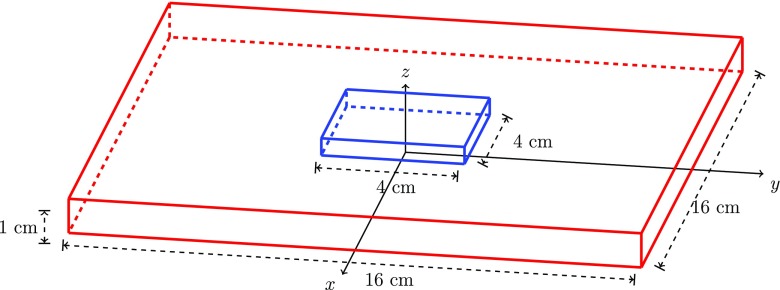
The tissue-blood model used in the simulations, showing the epicardium at *z* = 0, the endocardium at *z* = 1 and the central region, which is ischaemic tissue that extends part way from the endocardium towards the epicardium. Blood extends from the endocardium (that is, for *z* > 1)

In this work, we solved these equations in a rectangular block (16 cm × 16 cm × 1 cm) of tissue (Fig. 1), which used an (*x*,*y*,*z*) coordinate system, with the origin at the centre of the block, the epicardium at *z* = 0, the endocardium at *z* = 1 and a blood mass between *z* = 1 and *z* = 26 cm. An ischaemic region (4 cm × 4 cm with varying depths) that extended part way from the endocardium towards the epicardium was centred at the origin [27, 28].

While the depth (1 cm) of the slab was chosen to be representative of the thickness of the human left ventricular wall, the *x* and *y* dimensions (16 cm) and the depth of the blood (25 cm) were simply chosen so that the computational domain was large enough to allow the boundary potentials to approach zero [25, 27].

We chose to use a rectangular slab of ischaemic tissue because a study using the same model with a cylindrically shaped ischaemic region found that there was little difference in the EPDs for the two geometries [4, 5].

On the epicardium, we made the assumption that the cardiac fibres were aligned with the *x*-axis [25]. This defines the longitudinal direction of the cardiac tissue. Also in the plane of the epicardium, at right angles to this direction, we define the transverse direction of the cardiac tissue (along the *y*-axis). Mutually orthogonal to these two directions is the normal direction of the tissue (the *z*-axis). It was also assumed that fibre rotation varied linearly between the epicardium and the endocardium.

We represented the transmembrane potential *ϕ*_*m*_ during the ST segment in acute (phase I) ischaemia as follows [28, 61]

1 $$ \phi_{m}(x,y,z) = \Delta \phi_{p} \Psi(x) \Psi(y) \Psi(1-z) $$

where *ϕ*_*p*_ is the difference in plateau potentials between the normal and ischaemic tissue, and

2 $$ \Psi(t) = \left\{ \begin{array}{l} \frac{1-\exp(-a_{t}/ \lambda_{t}) \cosh(t/ \lambda_{t})}{1-\exp(-a_{t}/ \lambda_{t})}~~~~|t| \le a_{t}\\ \frac{\exp(-|t|/ \lambda_{t}) \sinh(a_{t}/ \lambda_{t})}{1-\exp(-a_{t}/ \lambda_{t})}~~~~~~~|t| > a_{t} \end{array} \right. $$

where *a*_*t*_ is the half-width of the ischaemic region for *t* = *x*,*y*,*z* and *λ*_*t*_ adjusts the steepness of the border zone between the normal and ischaemic zones. We set *ϕ*_*p*_ = −30 mV [21, 28], and *λ*_*t*_ = 0.01 ∀*t*, which resulted in a sharp interface between the two regions of tissue, and a narrow border zone, consistent with previous work [25, 28].

The same boundary conditions as in previous work [25] were used here: the edges of the tissue and the blood mass in the *x* and *y* directions as well as the epicardial surface are insulated; *ϕ*_*b*_ = 0 at the bottom of the blood; and the potential and current are continuous between the tissue and the blood.

The computational model used has 375,821 nodes and 360,000 non-uniformly spaced hexahedral finite volumes, with *x* and *y* resolutions on average of 2.5 mm and a *z* resolution of 0.2 mm, where the nodes are clustered around the borders of the ischaemic region. The solution technique, which is based on the finite volume method, has previously been verified [25]. We solved the governing equations using the various sets of parameters that will be discussed in the next section.

### Parameter ranges considered

A list of bidomain conductivity values from the literature is given in Table 1, where dashes indicate that values are not available, and information is given as to whether the study is experimental (E), partly experimental (PE) or theoretical (T). Additional information about the animal models used is also given for the experimental studies.

**Table 1:** Conductivity data (in mS/cm) from the indicated studies and the overall mean and standard deviation (std)

Study	Type	Notes	*g*_*il*_	*g*_*el*_	*g*_*it*_	*g*_*et*_	*g*_*in*_	*g*_*en*_
Bauer [8]	PE	Rabbit	0.65	–	0.042	–	0.033	–
Clerc [13]	E	Calf	1.7	6.3	0.19	2.4	–	–
Hand [18]	T		1.0	3.0	0.03	1.6	–	–
Hooks [19]	PE	Pig	2.6	2.6	0.26	2.5	0.08	1.1
Johnston [24]	T		1.9	3.2	0.35	2.2	0.08	1.2
Johnston [23]	T		2.4	2.4	0.35	2.0	0.08	1.1
Johnston [24]	T		3.1	2.0	0.35	2.2	0.08	1.2
Krassowska [33]	T		0.7	3.0	0.003	1.5	–	–
Le Guyader [34]	E	Dog	0.6	1.3	0.39	1.3	–	–
Le Guyader [35]	E	Dog	2.0	3.9	0.19	1.7	–	–
MacLachlan [39]	T		3.0	2.0	1.0	1.7	0.32	1.4
Roberts [46]	E	Dog	3.4	1.2	0.6	0.8	–	–
Roberts [45]	E	Dog	2.8	2.2	0.26	1.3	–	–
Roth [47]	T		3.5	3.0	0.3	1.8	–	–
Roth [49]	T		1.9	1.9	0.2	0.9	–	–
Stinstra [54]	T		1.6	2.1	0.05	0.6	–	–
Trayanova [60]	T		2.0	3.0	0.14	0.32	–	–
Mean			2.1	2.7	0.28	1.6	0.11	1.2
Std			0.9	1.2	0.24	0.6	0.1	0.1

Dashes in the table indicate that the values do not exist. Here, *α* = *g*_*il*_/*g*_*el*_. Information is given about the type of study, experimental (E), partly experimental (PE), theoretical (T) and, for the experimental studies, the animal model used

It is clear that there is considerable variation in the experimentally determined sets of four-conductivity values and that there are very few sets of six-conductivity values available. In practice, when four-conductivity sets are used in modelling studies, the assumption is made that the normal and transverse conductivities are equal. However, experiments have shown that this is not the case [11, 20].

For the purposes of this study, we aimed to consider the effect of varying the conductivity values over a wide, but plausible, range; that is, we wished to consider only sets of values that lead to physiologically reasonable conductivity ratios.

The final two rows in Table 1 give the mean and standard deviations for the datasets listed and we used these values to guide our choices for the data ranges for the conductivity values that are listed in Table 2. The means for *g*_*il*_ and *g*_*el*_ are 2.1 and 2.7 mS/cm, respectively, and since we decided to keep the ratio *g*_*il*_/*g*_*el*_ equal to 1 (see discussion in [23, 48]), we chose *g*_*il*_ = *g*_*el*_ = 2.4 mS/cm (the average of the two). We set *g*_*it*_ = 0.24 mS/cm, which is 0.1 of these values [48], which is consistent with its mean value of 0.28 mS/cm (given its large standard deviation). The remainder of the conductivities were taken to be *g*_*et*_ = 1.6 mS/cm, *g*_*in*_ = 0.1 mS/cm, *g*_*en*_ = 1.0 mS/cm, compared with their means from Table 1 of 1.6, 0.11 and 1.2 mS/cm, respectively.

**Table 2:** Data ranges for parameters used in this study, given in mS/cm for conductivities and degrees for fibre rotation (ROT)

Parameter	Minimum	Mean	Maximum
*g*_*il*_	1.2	*2.4*	3.6
*g*_*el*_	1.2	*2.4*	3.6
*g*_*it*_	0.12	*0.24*	0.36
*g*_*et*_	0.8	*1.6*	2.4
*g*_*in*_	0.05	*0.1*	0.15
*g*_*en*_	0.5	*1.0*	1.5
*g*_*b*_	3.25	*6.5*	9.75
ROT	60	*100*	140

Numbers in italic font indicate the inputs to which the outputs are most sensitive

In each of the six cases, we chose the range to be mean ± 50%, which meant that over all cases, except *g*_*it*_, an average of only two values from Table 1 did not lie in the chosen range. The values for *g*_*it*_ in the literature seem to be far less certain as they lie in the range 0.28 ± 0.24 mS/cm, with a maximum value of 1.0 mS/cm and a minimum value of 0.003 mS/cm. Given this, we decided to simply continue with the mean ± 50% formula used for the other conductivities, which meant that 10 out of 17 values from Table 1 lie in the chosen range.

The mean values in Table 2 give bulk conductivity ratios of *g*_*l*_/*g*_*t*_ = 2.6 and *g*_*t*_/*g*_*n*_ = 1.7, and conduction velocity ratios of *c*_*l*_/*c*_*t*_ = 2.4 and *c*_*t*_/*c*_*n*_ = 1.5, where $g_{A}/g_{B}=\frac {g_{iA}+g_{eA}}{g_{iB}+g_{eB}}$ and $c_{A}/c_{B}=\sqrt {(\frac {g_{iB}+g_{eB}}{g_{iB}g_{eB}})(\frac {g_{iA}g_{eA}}{g_{iA}+g_{eA}})}$. These values are in the same range as reported in previous studies (see [11, 20] and discussion in [23]).

In terms of conductivity ratios, in their study, Hopenfeld et al. [22] varied *g*_*il*_/*g*_*el*_ from 0.2–3, compared with our 0.3–3 (mean 1). For *g*_*il*_/*g*_*it*_, we use the range 3–30 (mean 10), compared with 20–50 [22] and 1–20 (mean 10) [43]. Lastly, we varied *g*_*el*_/*g*_*et*_ from 0.5–4.5 (mean 1.5), compared with 1–5 [22] and Potse et al. [43] who use the values 2.5 and 5.

The mean and ranges for the other parameters that were varied in this study, that is the conductivity of blood (*g*_*b*_) and fibre rotation angle (ROT), are also given in Table 2. Our choice of *g*_*b*_ in the range 6.5 ± 3.25 mS/cm was consistent with literature values, which include 2.39 mS/cm [39], 4 mS/cm [50], 6 mS/cm [43, 51] and 8 mS/cm [60]. Similarly, our choice of a fibre rotation angle from epicardium to endocardium of 100^∘^± 40^∘^ for the left ventricle was based on literature values of 120^∘^ (up to 180^∘^) [57], 140^∘^ [20], 112^∘^± 31^∘^ [3] and 103^∘^± 22^∘^ [59] in animals, as well as a recent study in ten human hearts that found a rotation from − 41^∘^(±26^∘^) to 66^∘^(±31^∘^) [38].

### Analysis methods

In order to consider the effect of uncertainty on the input parameters in the form of the EPD, we used three different methods of analysis: generalised polynomial chaos, Gaussian process emulators, and partial least squares regression coefficients. These were used to analyse the effect of uncertainty on the input parameters in various outputs that characterised the form of the EPD. We give details for each of the methods in the sections below.

#### Polynomial chaos

We implemented generalised polynomial chaos by allowing the various parameters that will be considered to vary uniformly across the ranges given in Table 2, using stochastic collocation [62, 63] with collocation points from a Clenshaw-Curtis numerical integration scheme. Then Smolyak’s method [52] was used to reduce the size of the integration space.

In this work, the six bidomain conductivities were varied and the other parameters were fixed to values that are detailed in the particular studies. For six input values, using level 2 integration results in *j* = 85 integration points (*g* *p* *q*(*j*),*p* = *i*,*e*,*q* = *l*,*t*,*n*) and integration weights *w*_*j*_. If we denote $\phi _{e}^{(j)}$ to be the extracellular potential distribution that results for each of these 85 sets of conductivities, the mean extracellular potential distribution is given by

$$\overline{\phi_{e}} = \sum\limits_{j=1}^{85} w_{j} \phi_{e}^{(j)} $$

and the standard deviation of the extracellular potential distribution is

$$(\phi_{e})_{\text{std}} = \left( \sum\limits_{j=1}^{85} w_{j} (\phi_{e}^{(j)}-\overline{\phi_{e}})^2 \right)^{\frac{1}{2}}. $$

The accuracy of this method was confirmed by calculating correlation coefficients and relative errors [23] between EPDs produced by level 2 and level 3 methods (the level 3 method requires 389 integration points). For example, for EPDs with the parameters 50% ischaemic depth, 120^∘^ fibre rotation, *g*_*b*_ = 6.7 mS/cm and mean values for the bidomain conductivities (Table 2), the correlation coefficient was 1.0 and the relative error was 1.2 × 10^−4^.

#### Gaussian process emulator

An emulator is a fast surrogate of a simulator, which in this case is the model that produces the EPDs (Section 2.1). Here, we constructed a Gaussian process (GP) emulator using a normal distribution to represent both the input variables and the outputs, with the result that both uncertainty quantification and sensitivity analysis could be performed using only a small number of design data (sets of inputs to the model).

Here, the design data were generated using a Latin hypercube (LHC) sampling routine that produced sets of input data by allowing each input variable to vary uniformly across the ranges (mean ± 50) in Table 2. This routine is in the software package GP_emu_UQSA (10.5281/zenodo.215521) developed at the University of Sheffield, which was used to construct the GP emulators, as well as to perform uncertainty quantification (UQ) and sensitivity analysis (SA).

For each set of input parameters generated by the LHC, the model from Section 2.1 was solved and an EPD was produced. These EPDs were analysed and a number of outputs (for example, the minimum potential), which characterise the EPD, were determined. Then, for each output, an emulator was fitted, using the GP_emu_UQSA software, to a (training) subset of the design data, using a Gaussian covariance function and a linear mean for the training.

In this work, 10% of the runs were kept as a test dataset for verification. The accuracy of the emulator fit was checked using the Mahalanobis distance [7], which compares the output of the emulator with the output of the simulator at the test points. This distance was calculated and compared with the mean and standard deviation of the appropriate reference distribution, which depends on the number of points in the training set and the number of input variables [12]. The final emulator was then built using the combined test and training dataset, according to methods that are detailed in Chang et al. [12] and in the documentation associated with the software.

Once each emulator was built, we used the software GP_emu_UQSA to produce main effect plots of the input variables against the mean effect for a particular output. The mean effect of an input variable *x*_*w*_ for a model *y* = *f*(**x**), where **x** are the input variables and *y* is the output, is defined to be [12] the conditional expectation of that output, conditional on the input variable (that is, after averaging over the remaining variables),

$$\text{mean~effect} = E\{f(x)|x_{w}\}. $$

For each emulator, the mean effect of the input variables was calculated in turn over the range *x*_*w*_ ∈ [0,1], with all the other input variables taken to be independently normally distributed with a mean of 0.5 and a variance of 0.04, which was chosen so that the input variables had a good coverage over [0,1].

In addition, we calculated main effect sensitivity indices to quantify the contribution of each input to each output [9, 42], again using the GP_emu_UQSA software. The main effect (sensitivity) index is defined [12] to be the ratio of the variance (Var) of the mean effect to the variance of the model output,

$$\text{sensitivity index} = \frac{\text{Var}[E\{f(x)|x_{w}\}]}{\text{Var}\{f(x)\}}. $$

This index does not take into account any variance in the outputs that could be due to interactions between the input variables, although the fact that this occurs can be inferred if the sum of the sensitivity indices is less than 1. Note, also, that because the sensitivity index involves variances, it can never be negative and is therefore unsigned, unlike the PLS coefficients discussed below.

#### Partial least squares regression

The third method of analysis we used again involved the construction of an emulator using the design data from Section 2.3.2, this time by partial least squares (PLS) regression. In this case, the outputs were regressed against the input variables using the PLS approach as implemented in the NIPALS algorithm [1, 16] and described in the study by Sobie [53]. PLS regression is a generalisation of principal component analysis, in that it searches for a set of components that simultaneously decompose both the input and output vectors under the constraint that the components should explain the maximum amount possible of the covariance between the inputs and outputs [1]. This approach again allowed us to assess the relative effects of various input variables, with each coefficient indicating the change that would occur in a model output when an input variable is increased or decreased.

## Results

### Polynomial chaos

**Fig. 2:**
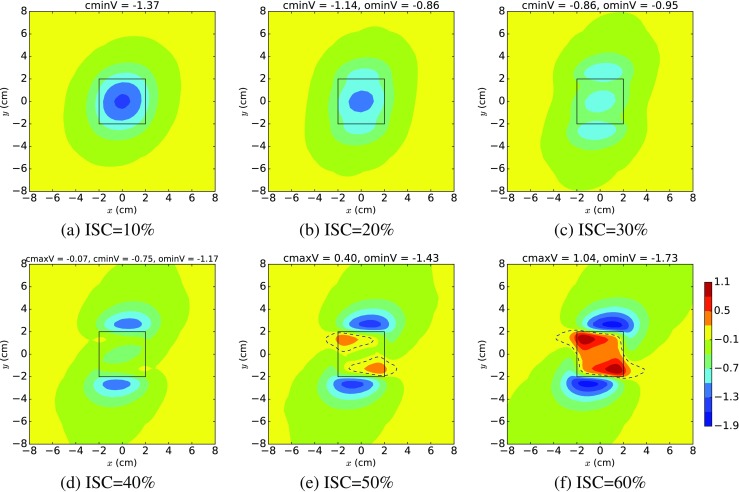
Polynomial chaos mean epicardial potential distributions (in mV) for various values of ischaemia, produced with *g*_*b*_ = 6.5 mS/cm, fibre rotation = 100^∘^ and varying the conductivities across the ranges given in Table 2. The solid line indicates the central ischaemic region and the dashed line is the zero of potential. The headings cminV, cmaxV and ominV give the values for the minimum (minV) and maximum (maxV) potentials (in mV) over either the central (c) ischaemic region or outside (o) this region

Figure 2 shows a set of mean EPDs, as the depth of ischaemia increased from 10 to 60%, which we constructed using generalised polynomial chaos (Section 2.3.1), by fixing *g*_*b*_ = 6.5 mS/cm and ROT = 100^∘^ at their mean values (Table 2), and allowing the six bidomain conductivity values (*g*_*pq*_,*p* = *i*,*e*,*q* = *l*,*t*,*n*) to vary uniformly across their parameter ranges (Table 2). This then gave a set of ‘representative’ EPDs for ischaemic depths (ISC) of 10–60% (Fig. 2a–f).

There are clear qualitative differences in the EPDs in Fig. 2, with the pattern changing from one central minimum (a), through (b) to a tripole pattern of three minima (c), followed by the development of two maxima in the central region (that is, above the ischaemic region), flanked by two minima along the lateral boundaries of the central region (e)–(f).

Quantitative differences between the EPDs can be observed in the values given for cminV and cmaxV (minimum and maximum potential in the central (c) region, respectively) and ominV (minimum potential in the region outside (o) the central region). The headers of plots (a) and (b) indicate that the EPDs for 10 and 20% ischaemia have a global minimum in the central region (cminV < ominV). However, as the ischaemic depth increases to 30 and 40%, the global minimum occurs outside the central region (ominV < cminV), even though there is still a minimum in the central region (cminV < 0). Finally, for ischaemic depths of 50 and 60%, ST elevation over the central region develops (cmaxV > 0), with two minima along the lateral boundaries of the central region.

From now on, we will refer to the scenario with a global minimum in the central region (e.g. Fig. 2a, b) as ST depression (type 1) and the scenario with the tripole of minima where the central minimum is no longer the global minimum (e.g. Fig. 2c, d) as ST depression (type 2). The final category, ST elevation, is exemplified by Fig. 2e, f and involves two maxima in the central region, flanked by two minima.

From the header values, we observe that, as the ischaemic depth increases from 10 to 40%, cminV increases from − 1.37 to − 0.75 mV, and then if we consider cmaxV, this continues to increase from − 0.07 mV at 40% ischaemia to 1.04 mV at 60%. This increase in the value of the potential in the centre region is paralleled by a strengthening of the minima on the boundaries, where ominV decreases from − 0.95 mV at 30% ischaemia to − 1.73 mV at 60% ischaemia.

**Fig. 3:**
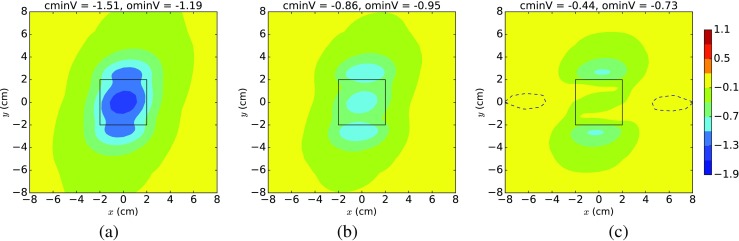
Polynomial chaos mean epicardial potential distributions, produced with *g*_*b*_ = 6.5 mS/cm, fibre rotation = 100^∘^, ischaemia = 30% and varying the conductivities across the ranges given in Table 2, showing **a** mean - std, **b** mean and **c** mean + std. Definitions are given in Fig. 2

Using the standard deviations produced by the polynomial chaos approach, we constructed sets of three EPDs using the data from Fig. 2. In these sets of plots (Fig. 3 is an example for ischaemia = 30%), the first and last plots used values one standard deviation (std) below and above the mean, respectively, and the middle one was the mean EPD from Fig. 2. We found that, in many cases, moving one standard deviation from the mean led to an EPD of a different character. For example, in Fig. 3, the EPD one std below the mean (a) now exhibits ST depression (type 1), with cminV < ominV, rather than ST depression (type 2) as in (b) and (c), where ominV < cminV < 0.

**Fig. 4:**
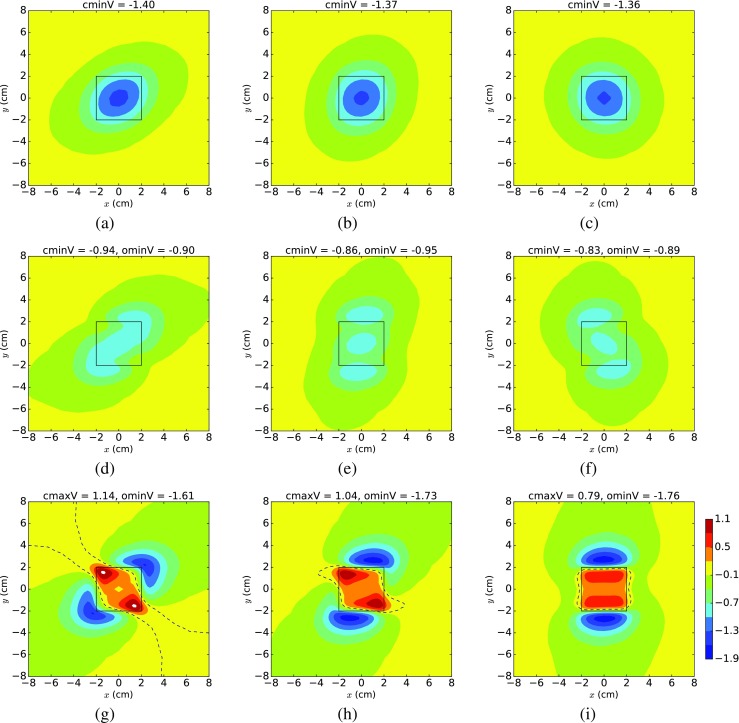
Polynomial chaos mean epicardial potential distributions, produced with *g*_*b*_ = 6.5 mS/cm and by varying the conductivities over the ranges in Table 2. From left to right across each row, fibre rotation is 60^∘^, 100^∘^ and 140^∘^. The top row is 10% ischaemia, the middle row 30% and the bottom row 60% ischaemia. Definitions are given in Fig. 2

Since we produced the plots in Figs. 2 and 3 with a fixed value of 100^∘^ for ROT, we also produced an additional set of polynomial chaos mean EPDs (Fig. 4), where we set ROT to its minimum (60^∘^), mean (100^∘^) and maximum (140^∘^) values. We used these three values for ROT across each row in Fig. 4, where the rows correspond to ischaemic depths of 10% (top), 30% (middle) and 60% (bottom). These depths were chosen because they are representative of the three scenarios: ST depression (type 1), ST depression (type 2) and ST elevation, respectively. It can be seen from Fig. 4 that increasing the degree of fibre rotation (i.e. across a row) results in an anticlockwise rotation of the EPDs, for all the ischaemic depths.

### Possible EPDs for 30% ischaemia

**Fig. 5:**
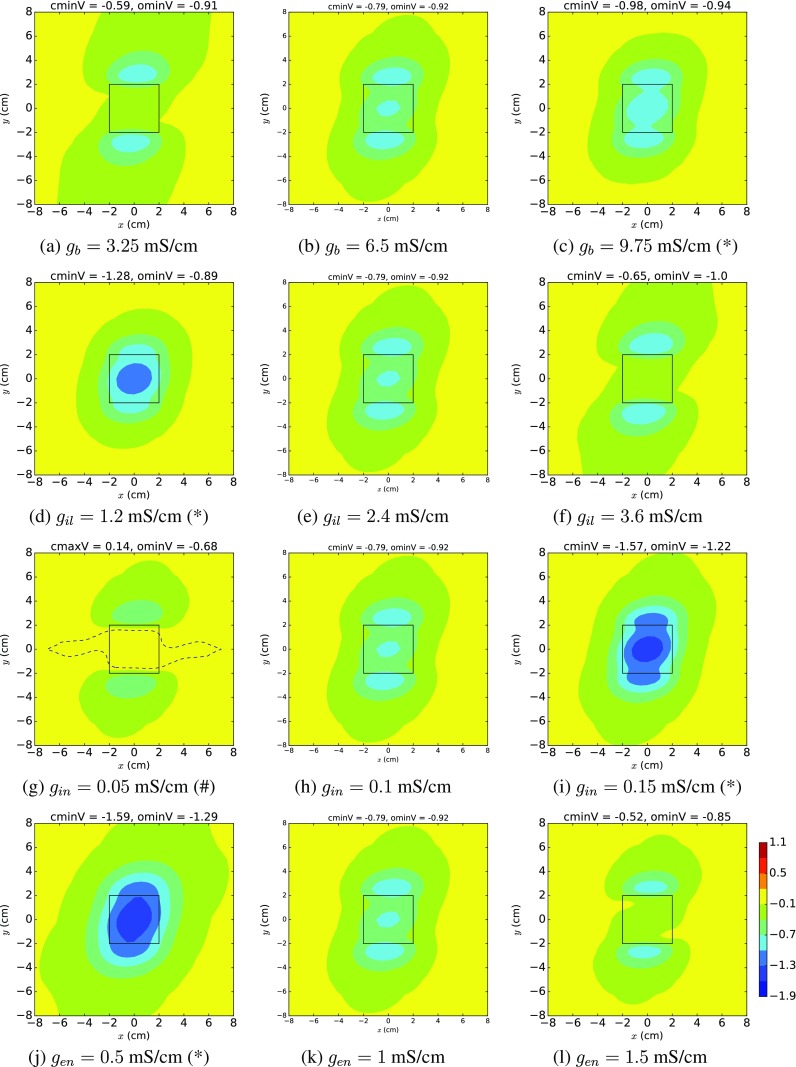
Epicardial potential distributions, where each row uses the minimum, mean and maximum of a particular parameter’s values, for 30% ischaemia and mean values for all other variables. Definitions are given in Fig. 2. Distributions marked with asterisk exhibit ST depression (type 1) behaviour, those marked with number sign show ST elevation and the remainder demonstrate ST depression (type 2) behaviour

The polynomial chaos mean EPDs in Fig. 4d–f show that the ‘average’ EPD corresponding to 30% ischaemia is the three minima tripolar pattern of ST depression (type 2). However, this is not always the case for 30% ischaemia, as is hinted at in Fig. 3a and will be shown in Fig. 5. The plots in Fig. 5 are not polynomial chaos plots but are, instead, produced by setting all but one of the variables to the means given in Table 2 and allowing the other variable to range from its minimum, to its mean and then to its maximum across each row.

Plots are presented in Fig. 5 only for those variables (*g*_*b*_,*g*_*il*_,*g*_*in*_,*g*_*en*_) where there is a change in the qualitative behaviour from that of the mean scenario, which is ST depression (type 2). For example, for *g*_*in*_ in the third row from the top, examination of the EPD patterns and the header values reveals that the minimum value for *g*_*in*_ (along with the means for all the other variables) results in ST elevation (g), while the maximum value for *g*_*in*_ leads to ST depression (i).

In Fig. 5, it can be seen that ST depression (type 1), indicated with an (*), develops for high *g*_*b*_ and *g*_*in*_ values and low *g*_*il*_ or *g*_*en*_ values. On the other hand, low *g*_*in*_ leads to ST elevation (indicated with a (#)). These results demonstrate that all three of the basic scenarios, ST depression (type 1), ST depression (type 2) and ST elevation, can occur for 30% ischaemia, depending on the values of either the blood conductivity or certain of the bidomain conductivities (*g*_*il*_,*g*_*in*_,*g*_*en*_).

To test this still further, we produced additional EPDs with mean values (Table 2) for all input variables, except for *g*_*il*_,*g*_*in*_ and *g*_*en*_. We set these to *g*_*il*_ = 1.2 mS/cm, *g*_*en*_ = 0.5 mS/cm, and *g*_*in*_ = 0.15 mS/cm, that is the minimum values for the first two and the maximum value for *g*_*in*_. We chose those, based on Fig. 5, as being the most likely to result in ST depression. We found that, even with ischaemic depths up to 90%, it was still possible to achieve ST depression (type 1) using these three values.

The realisation that, for most ischaemic depths, any one of the three basic scenarios could occur, then led us to the decision that it does not make sense to analyse the effect of the input parameters at various different ischaemic depths. We decided, instead, to focus on the effect on EPDs that fall into one of the three basic categories: ST depression (types 1 and 2) and ST elevation. These analyses will be presented in the following subsections.

### ST depression (type 1) (cminV < ominV < 0)

Here, we consider the ST depression (type 1) EPDs, which are characterised by the global minimum occurring above the central ischaemic region (Fig. 2a). There may also be two other minima that occur outside this region, provided that cminV < ominV (Fig. 5i). These EPDs have a characteristic ‘ellipse’-type shape.

In this work, the EPDs were analysed using four outputs that were drawn from the EPD. The first three were potentials, defined in Section 3.1, at various positions: cminV, cmaxV and ominV. We included the outputs cmaxV and ominV partly for consistency with later work and partly because ST depression (type 1) EPDs may also look like Fig. 5i, where there are outer minima and thus ominV is important. The fourth is the orientation of the ‘ellipse’ that we fitted to the contour plot; that is, the angle between the semi-major axis of the ellipse and the positive *x*-axis.

We produced the design data for this analysis using a 10% ischaemic depth and by varying all the other eight input variables (ROT, *g*_*b*_,*g*_*il*_,*g*_*el*_,*g*_*it*_,*g*_*et*_,*g*_*in*_,*g*_*en*_) uniformly across their data ranges (Table 2). Using the 250 sets of input variables generated by the LHC sampling, we produced EPDs, of which 247 were of the type ST depression (type 1). We used the first 240 of these in the analysis presented below. This reduction to 240 sets of input variables and outputs was necessary to divide the design data into sensible sized training (216) and validation (24) sets for constructing the GP emulator (here 90 and 10%, respectively), a limitation that was in the GP_emu_UQSA software at the time. We found that the reduction from 247 to 240 sets was not significant in terms of the results and we will discuss this further, below.

**Fig. 6:**
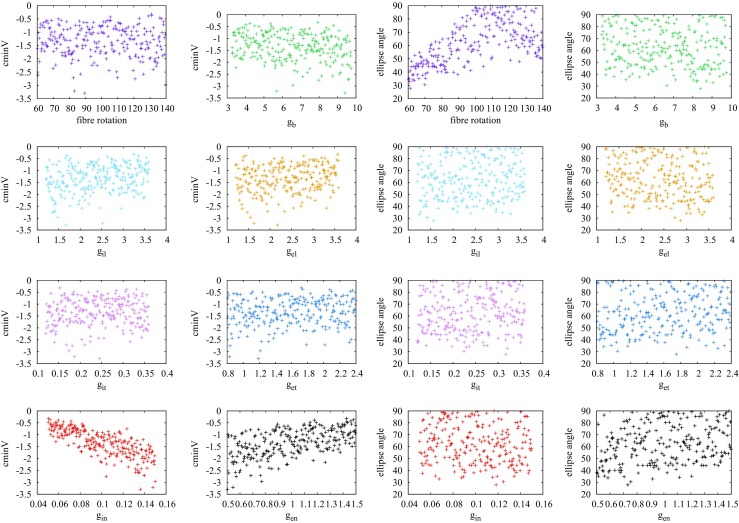
Design data for the features centre minimum voltage (cminV), in mV, and ellipse angle, in degrees, of the epicardial potential distributions of ST depression (type 1). These are plotted against each of the eight input variables, with units of mS/cm for conductivities and degrees for fibre rotation

We began by using the model (simulator) to produce an EPD for each of the 240 sets of input variables and then used these EPDs to calculate the outputs cminV, cmaxV, ominV and ellipse angle in each case. These design data are plotted in Fig. 6, for two of the input variables, with cminV plotted in the two left-hand columns and ellipse angle in the two right-hand columns. The data for ominV and cmaxV are reasonably similar to those for cminV, except for magnitudes, and can be found in the Supplementary material (Fig. 1).

We fitted emulators for each of cminV, cmaxV, ominV and ellipse angle, using the design data, as described in Section 2.3.2. We then used these emulators to produce main effect plots, by allowing each input variable to vary across the (normalised) range 0–1, while the other inputs were fixed at 0.5 (the mean) with a variance of 0.04. So in each case, the distribution for an input is taken to have the same mean as chosen in Table 2, with a standard deviation of 0.2, giving, for example, for *g*_*il*_ and *g*_*el*_ (in unscaled units), the input distribution 2.4 ± 0.48 mS/cm.

**Fig. 7:**
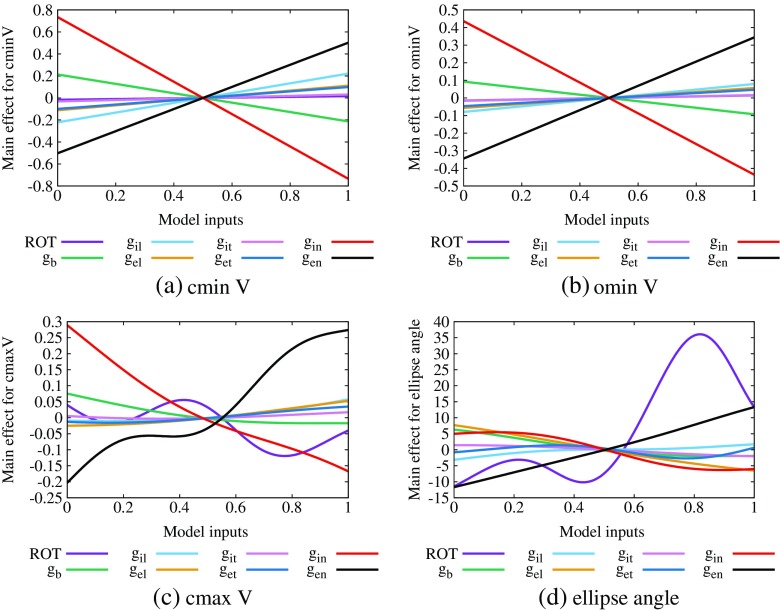
Main effect plots for outputs of EPDs showing ST depression (type 1), **a** cminV, **b** ominV, **c** cmaxV and **d** ellipse angle. Plots are produced by allowing each input to vary over the normalised range [0,1], while all other inputs are fixed at a mean of 0.5 with a variance of 0.04

The main effect plots, which show the change in the expectation of the output, across the input range, for cminV, cmaxV, ominV and ellipse angle are given in Fig. 7a–d, respectively. In each case, 0 on the vertical scale represents the emulator mean value for the particular variable (− 1.35 mV for cminV, − 0.49 mV for cmaxV, − 0.89 mV for ominV and 58.2^∘^ for ellipse angle).

Figure 7a shows that the input variables that have the most effect on cminV are *g*_*in*_ and *g*_*en*_ and that their effects are opposite to one another; that is, increasing *g*_*in*_ decreases cminV and vice versa for *g*_*en*_. The plots for cmaxV and ominV show very similar trends. In the case of the ellipse angle, Fig. 7d indicates that increasing fibre rotation has a positive effect on ellipse angle (that is, the ellipse rotates anticlockwise). This is consistent with Fig. 4a–c.

To quantify these effects, we produced sensitivity indices associated with each of our emulators, using means of 0.5 and variances of 0.02 for each input variable. These indices quantify the contribution of the variance in each input to the variance in the output and they are given in the top half of Table 3. The sensitivity indices showed that cminV was sensitive to both normal conductivities, with a value for *g*_*in*_ of 0.5 and for *g*_*en*_ of 0.23, and that cminV was not sensitive to any of the other input variables. The results for *g*_*in*_ were 0.44 for cmaxV and 0.47 for ominV and for *g*_*en*_ 0.47 for cmaxV and 0.29 for ominV. In the case of the ellipse angle, fibre rotation was the only variable to which it was sensitive (0.66).

**Table 3:** Sensitivities of the outputs cminV and ellipse angle to the inputs listed, for EPDs exhibiting ST depression (type 1)

Outputs	ROT	*g*_*b*_	*g*_*il*_	*g*_*el*_	*g*_*it*_	*g*_*et*_	*g*_*in*_	*g*_*en*_
cminV	0.00	0.04	0.05	0.01	0.00	0.01	*0.50*	*0.23*
cmaxV	0.20	0.01	0.01	0.01	0.00	0.00	*0.29*	*0.46*
ominV	0.00	0.02	0.02	0.01	0.00	0.01	*0.47*	*0.29*
Ellipse angle	*0.66*	0.01	0.00	0.02	0.00	0.01	0.05	0.06
cminV	0.02	− 0.22	0.23	0.11	0.03	0.10	−*0.74*	*0.51*
cmaxV	− 0.18	− 0.13	0.13	0.05	0.00	− 0.02	−*0.65*	*0.64*
ominV	0.03	− 0.16	0.13	0.09	0.03	0.08	−*0.73*	*0.57*
Ellipse angle	*0.59*	− 0.14	0.08	− 0.14	0.04	0.10	− 0.20	0.24

(Top half of table) GP emulator sensitivity indices and (bottom half of table) partial least squares coefficients. Note that the GP sensitivity indices are unsigned

Numbers in italic font indicate the inputs to which the outputs are most sensitive

The total of the cminV indices is 0.85, for cmaxV 0.98 and ominV 0.82, while that of the ellipse angle indices is 0.82. This indicates that most of the variance in the outputs is explained by variance in the input variables individually.

We also analysed the design data (Fig. 5) using a partial least squares (PLS) approach [1, 53]. This produces regression coefficients that indicate the way each output changes with each input; that is, if the effect of the input variable is positive, then the output increases and vice versa for a negative coefficient. We note that this ‘directionality’ is an advantage of PLS over GP emulators, where the sensitivity indices are unsigned. We used the same 240 points to calculate the PLS coefficients in the bottom half of Table 3 as we used to construct the GP emulator. We also repeated the PLS analysis for the original 247 points and we found that any differences in the results were extremely minor (in the second decimal place).

Despite the different approaches, and the differences in the magnitudes of the GP emulator sensitivity indices and the PLS coefficients, they are consistent with one another and with those in Fig. 7. We see again that cminV, cmaxV and ominV are most sensitive to *g*_*in*_ (negative effect) and *g*_*en*_ (positive effect), with *g*_*in*_ having the stronger effect, and that the ellipse angle is most sensitive to the fibre rotation angle (positive effect).

Finally, it should be noted that the magnitudes of the coefficients produced by the PLS approach should only be considered in relation to the other coefficients for that particular output variable and not in a global sense [53]. That is, in row 1 of Table 4 we see that the variance in cminV is primarily explained by *g*_*in*_ and *g*_*en*_ because the absolute values of their coefficients are considerably larger than those of the remaining input variables.

**Table 4:** Sensitivities of the outputs ominV, cminV, cmaxV and angmin to the inputs listed, for EPDs exhibiting ST depression (type 2)

Outputs	ROT	*g*_*b*_	*g*_*il*_	*g*_*el*_	*g*_*it*_	*g*_*et*_	*g*_*in*_	*g*_*en*_
ominV	0.00	0.00	0.06	*0.13*	0.01	0.06	*0.55*	0.09
cminV	0.00	0.03	0.04	0.01	0.00	0.01	*0.56*	*0.24*
cmaxV	0.00	0.03	*0.11*	0.00	0.00	0.00	*0.33*	*0.28*
angmin	*0.84*	0.00	0.00	0.00	0.00	0.00	0.01	0.01
ominV	0.02	− 0.02	− 0.26	*0.42*	0.08	0.27	−*0.73*	0.32
cminV	0.04	− 0.31	0.32	0.20	0.09	0.14	−*1.23*	*0.85*
cmaxV	− 0.13	− 0.44	*0.74*	− 0.18	− 0.01	− 0.13	−*1.27*	*1.23*
angmin	*0.97*	− 0.05	0.07	− 0.02	− 0.01	− 0.02	− 0.09	0.13

(Top half of table) GP emulator sensitivity indices and (bottom half of table) partial least squares coefficients

Numbers in italic font indicate the inputs to which the outputs are most sensitive

### ST depression (type 2) (ominV < cminV < 0)

The next category of EPDs that we will analyse is those where there is a characteristic tripole pattern of three depressions, one of which is over the central region. This central depression is typically flanked by two minima outside the central region (Fig. 2c, d). In this case, ominV < cminV so that the global minimum value is outside the central region.

As mentioned in Section 3.2, variations in the input conductivity values can lead to all three cases for the form of the EPD when the ischaemic depth is around 30–40%. So to produce approximately 200 EPDs of the tripolar type, we used the LHC routine to produce 750 datasets by setting the ischaemic depth to 30% and varying the other eight input variables uniformly across their ranges (Table 2). This produced 292 EPDs (39%) of ST depression (type 1), 222 EPDs (30%) of ST depression (type 2) and 236 EPDs (31%) showing ST elevation. We took only the first 220 of the 222 ST depression (type 2) EPDs as our design data (for similar reasons to those in Section 3.3) and identified a number of outputs that could be used to characterise the EPDs.

We are interested in the values for the minima, both inside the central region (cminV) and outside the central region (ominV), as well as the maximum value in the central region (cmaxV). In addition, we are not so much interested in the orientation of the EPD as in Section 3.3, but rather, we are interested in the position of the outside minimum. These two things are not the same in all cases as sometimes the central minimum is not at (0,0). So, we report the position of the minimum, which we will designate as angmin, by finding the angle to the positive *x*-axis of a line from (0,0) to the minimum that occurs in the *y* > 0 half-plane. We note that symmetry in the model about the *x*-axis results in the same potential value for each of the outside minima.

**Fig. 8:**
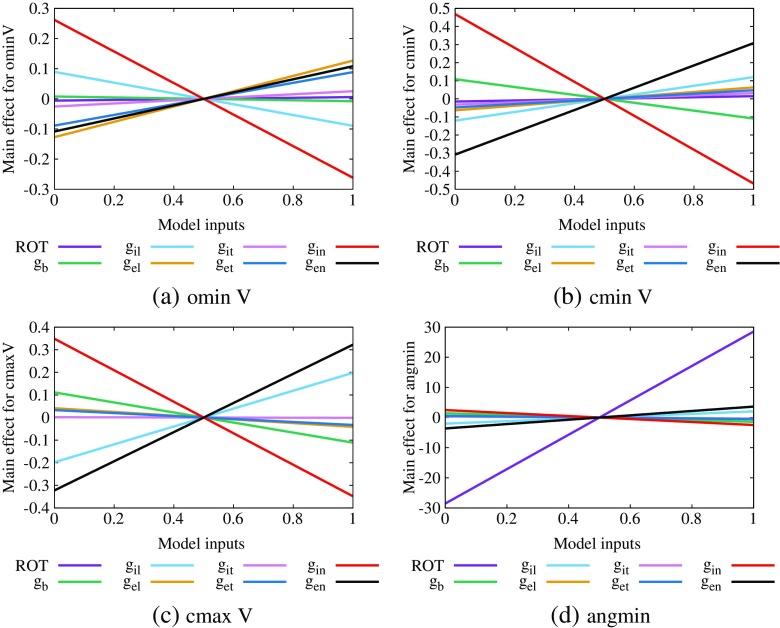
Main effect plots for outputs of EPDs showing ST depression (type 2), **a** ominV, **b** cminV, **c** cmaxV and **d** angmin. Plots are produced by allowing each input to vary over the normalised range [0,1], while all other inputs are fixed at a mean of 0.5 with a variance of 0.04

We first extracted these four outputs from the EPDs that correspond to each of the 220 input datasets and then plotted the design data in Supplementary Figs. 2 and 3, with ominV in the two left columns and cminV in the two right columns of Supplementary Fig. 2 and similarly for cmaxV and the angmin in Supplementary Fig. 3. Then following the approach of Section 3.3, we analysed the data by constructing GP emulators for each of the four outputs and produced main effect plots (Fig. 8) and sensitivity indices (Table 4). In this case, the emulator mean values were ominV (− 0.91 mV), cminV (− 0.81 mV), cmaxV (− 0.25 mV) and angmin (83.6^∘^).

The values in Table 4 indicate that the input variables that have the greatest effect on the EPD potentials, both above the ischaemic region (cminV and cmaxV) and outside it (ominV), are *g*_*in*_ and *g*_*en*_, again acting in opposite directions, with increased *g*_*in*_ related to decreased potential and vice versa for *g*_*en*_ (Fig. 8). In addition, ominV is slightly sensitive to *g*_*el*_ and less sensitive to *g*_*en*_ and cmaxV is slightly sensitive to *g*_*il*_.

In a similar fashion to Section 3.3, we also see that the angle of the outside minimum is sensitive to the degree of fibre rotation and that increasing fibre rotation leads to an increase in the angle of the minimum; that is, the minimum moves anticlockwise (and hence so does the tripole). The design data where fibre rotation is plotted against angmin in Supplementary Fig. 3 (top row, third column) not only shows this relationship but it also shows that the angle data are discrete and stepped; however, this appears to be an artefact of the mesh spacing.

Once again, the PLS coefficients that are found using the same design data (Table 4, bottom half) are consistent with the results that we have just presented.

### ST elevation (cmaxV > 0)

The final category of EPDs is ST elevation, with a central maximum (or two central maxima) over the ischaemic region, flanked by two minima near the boundaries of the central region (Fig. 2e, f). In this case, cmaxV is the global maximum and ominV is the global minimum.

To make the design data, we took the ischaemic depth to be 60% and produced 250 EPDs by using the sets of eight input variables that came from the LHC routine when the eight input variables were varied uniformly across their ranges (Table 2). Of these 250 EPDs, 227 were of the ST elevation type and so the first 220 of these were used for the analysis (once again the reduction did not affect the results).

Clearly, cmaxV and ominV are important outputs in this case, as well as the positions of the maxima (angmax) and minima (angmin). We quantified the latter two by calculating, as in Section 3.4, the angle a line drawn from (0,0) to the maximum or minimum in the *y* > 0 half-plane makes with the positive *x*-axis. Then, we plotted these design data against the eight input variables, cmaxV and ominV in Supplementary Fig. 4 and incomes and angmin in Supplementary Fig. 5.

**Fig. 9:**
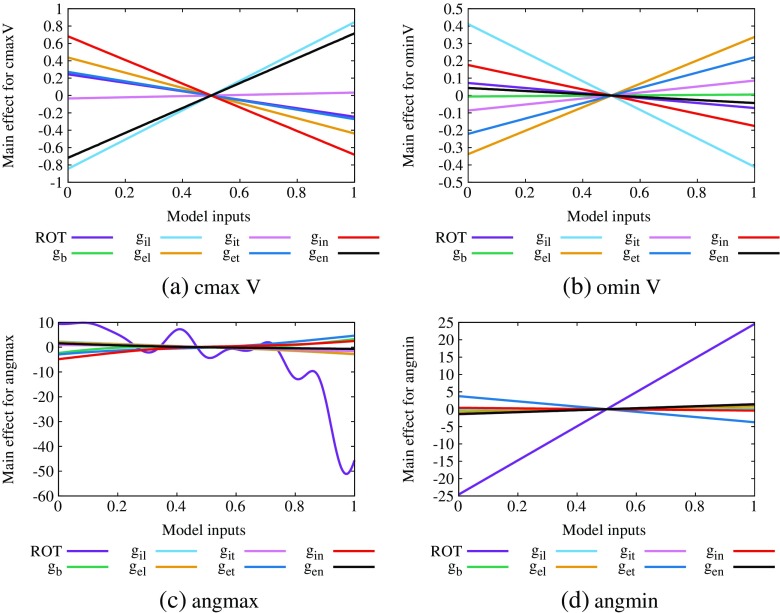
Main effect plots for outputs of EPDs showing ST elevation, **a** cmaxV, **b** ominV, **c** angmax and **d** angmin. Plots are produced by allowing each input to vary over the normalised range [0,1], while all other inputs are fixed at a mean of 0.5 with a variance of 0.04

Figure 9 shows the main effect plots, for cmaxV, ominV, angmax and angmin, produced when we fitted GP emulators to their design data. Table 5 gives the sensitivity indices and the corresponding PLS indices. Here, the emulator mean values are cmaxV (1.11 mV), ominV (− 1.72 mV), angmax (129.5^∘^) and angmin (67.7^∘^).

**Table 5:** Sensitivities of the outputs cmaxV, ominV, angle of the maximum and angle of the minimum to the inputs listed, for EPDs exhibiting ST elevation

Outputs	ROT	*g*_*b*_	*g*_*il*_	*g*_*el*_	*g*_*it*_	*g*_*et*_	*g*_*in*_	*g*_*en*_
cmaxV	0.03	0.03	*0.30*	0.08	0.00	0.03	*0.19*	*0.22*
ominV	0.01	0.00	*0.38*	*0.25*	0.02	0.11	0.07	0.00
angmax	*0.49*	0.03	0.01	0.01	0.01	0.03	0.03	0.01
angmin	*0.84*	0.00	0.00	0.00	0.00	0.02	0.00	0.00
cmaxV	− 0.19	− 0.21	*0.63*	− 0.34	0.03	− 0.21	−*0.52*	*0.54*
ominV	− 0.12	0.01	−*0.64*	*0.55*	0.14	0.36	− 0.28	− 0.07
angmax	−*0.49*	0.10	− 0.19	− 0.12	0.00	0.17	0.17	− 0.15
angmin	*0.97*	0.01	− 0.01	0.04	0.06	− 0.15	− 0.01	0.05

(Top half of table) GP emulator sensitivity indices and (bottom half of table) partial least squares coefficients

Numbers in italic font indicate the inputs to which the outputs are most sensitive

This time the results for cmaxV are similar to those for cminV in Sections 3.3 and 3.4; that is, cmaxV is sensitive to changes in *g*_*in*_ and *g*_*en*_ and these inputs have opposite effects, in that increasing *g*_*in*_ means decreasing cmaxV and vice versa for *g*_*en*_ (see Fig. 9a and Table 5). However, in this case, the variable to which cmaxV is most sensitive is *g*_*il*_ (and this is a positive relationship like *g*_*en*_).

Conversely, increasing *g*_*il*_ results in a decrease in ominV and it is *g*_*en*_ which has the positive relationship with ominV (see Fig. 9b and Table 5). Also we see that neither *g*_*in*_ nor *g*_*en*_ has a significant effect on ominV.

As was observed in Section 3.4, the results in Fig. 9c and Table 5 show that both angmax and angmin are related to the fibre rotation angle, with a strong positive relationship in the case of angmin (that is, as the fibre rotation angle increases the minimum moves anticlockwise).

The relationship for angmax is more complicated, as can be seen in the plot for angmax against ROT (Supplementary Fig. 5, top row, column 1). This shows that the maximum is consistently at an angle of approximately 130–140^∘^ for all fibre rotation angles up to about 120^∘^ and then, after that, there are some cases where there is a marked drop in the size of the angles. This situation is illustrated in Fig. 4h, i, where fibre rotation changes from 100^∘^ to 140^∘^ and the *y* > 0 maximum moves from the top left corner of the central region across to the right. This non-linear relationship between ROT and angmax is responsible for the non-linear fit of the emulator (Fig. 9c).

In all cases but angmax, the sum of the GP emulator sensitivity indices is around 0.85, which indicates that most of the output variance can be explained by the individual variance in the input variables. This is not the case for angmax where the sum is 0.61, perhaps because the emulator is not such a good fit for the data.

### Summary of results

We have summarised the results from Sections 3.3, 3.4 and 3.5 in Table 6, where the input variables that have a significant effect on the outputs are marked by arrows. In this table, *↑* indicates that increasing the input variable results in an increase in the output and *↓* indicates the opposite effect.

**Table 6:** Summary of the outputs (columns) and the input variables (rows) to which they are sensitive

	ROT	*g*_*b*_	*g*_*il*_	*g*_*el*_	*g*_*it*_	*g*_*et*_	*g*_*in*_	*g*_*en*_
ST depression (type 1)								
cmaxV							*↓*	*↑*
cminV							*↓*	*↑*
ominV							*↓*	*↑*
Ellipse angle	*↑*							
ST depression (type 2)								
cmaxV			*↑*				*↓*	*↑*
cminV							*↓*	*↑*
ominV				*↓*			*↓*	
angmin	*↑*							
ST elevation								
cmaxV			*↑*				*↓*	*↑*
ominV			*↓*	*↑*				
angmax	*↓*							
angmin	*↑*							

Blank spaces indicate no significant relationship. An increase in the input variable that results in an increase in the output is represented by an upward pointing arrow and an increase in the input variable that results in a decrease in the output is represented by a downward pointing arrow

One thing that is immediately apparent from Table 6 is that none of the outputs that have been chosen to characterise the EPDs is sensitive to *g*_*b*_,*g*_*it*_ or *g*_*et*_. We can also see that the orientation of the EPD and position of the maximum or minimum are affected only by the degree of fibre rotation and that the effect is opposite for the angmin or angmax, in the case of ST elevation. Various outputs are sensitive to changes in *g*_*il*_,*g*_*el*_,*g*_*in*_ or *g*_*en*_ and, where both *g*_*ip*_ and *g*_*ep*_ (*p* = *l* or *n*) are involved, they have an opposite effect.

### Effect of conductivity ratios

Since some previous studies [22, 43] have considered the effect of conductivity ratios on ST depression, rather than conductivity values, the final table in this work (Table 7) will present values for PLS correlations between various conductivity ratios and the voltage outputs from Table 6.

**Table 7:** Partial least squares coefficients indicating the correlation between various conductivity ratios and some outputs associated with the indicated types of epicardial potential distributions

		cmaxV	cminV	ominV		cmaxV	cminV	ominV
ST depression	*g*_*il*_/*g*_*el*_	0.05	0.08	0.03	*g*_*il*_/*g*_*it*_	− 0.19	− 0.21	− 0.24
(type 1)	*g*_*it*_/*g*_*et*_	0.01	− 0.08	− 0.07	*g*_*il*_/*g*_*in*_	*0.67*	*0.78*	*0.73*
	*g*_*in*_/*g*_*en*_	−*0.94*	−*0.90*	−*0.95*	*g*_*el*_/*g*_*et*_	0.28	0.14	0.18
					*g*_*el*_/*g*_*en*_	−*0.50*	− 0.33	− 0.41
ST depression	*g*_*il*_/*g*_*el*_	0.28	− 0.16	− 0.44	*g*_*il*_/*g*_*it*_	− 0.19	−*0.43*	−*0.49*
(type 2)	*g*_*it*_/*g*_*et*_	0.03	− 0.05	− 0.11	*g*_*il*_/*g*_*in*_	*0.64*	*0.51*	0.21
	*g*_*in*_/*g*_*en*_	−*0.88*	−*0.74*	−*0.54*	*g*_*el*_/*g*_*et*_	0.40	0.20	0.07
					*g*_*el*_/*g*_*en*_	−*0.77*	− 0.22	0.15
ST elevation	*g*_*il*_/*g*_*el*_	*0.64*	− 0.19	−*0.80*	*g*_*il*_/*g*_*it*_	0.06	− 0.38	−*0.53*
	*g*_*it*_/*g*_*et*_	0.18	0.09	− 0.21	*g*_*il*_/*g*_*in*_	*0.72*	*0.46*	0.03
	*g*_*in*_/*g*_*en*_	−*0.70*	−*0.49*	− 0.18	*g*_*el*_/*g*_*et*_	0.19	0.25	− 0.02
					*g*_*el*_/*g*_*en*_	−*0.64*	− 0.11	*0.46*

Numbers in italic font indicate the inputs to which the outputs are most sensitive

The correlation results for the ratios (*g*_*iq*_/*g*_*eq*_,*q* = *l*,*t*,*n*) in the left column of Table 7 are related to the results in Table 6, but those in Table 7 provide extra information about the strength of the relationships. In Table 7, we see again that the transverse ratio *g*_*it*_/*g*_*et*_ is not significantly correlated with any of the outputs in any of the cases. We also see that for all three types of EPDs, *g*_*in*_/*g*_*en*_ is strongly negatively correlated with all of cmaxV, cminV and ominV, except for ominV in the ST elevation case, and that the normal ratio is the only significant ratio in the two ST depression cases. For the ST elevation case, significant correlations are also found between *g*_*il*_/*g*_*el*_ and cmaxV and ominV, the first of which is a positive correlation and the second is negative.

The right-hand column of Table 7 contains PLS correlations for the anisotropy ratios (*g*_*pl*_/*g*_*pt*_ and *g*_*pl*_/*g*_*pn*_, *p* = *i*,*e*) with the same outputs. The longitudinal to transverse versions of these ratios were considered in previous studies [22, 43], but here, we also include the longitudinal to normal ratios, since we are using six and not four bidomain conductivities.

### Position of the minimum

We saw in Table 6 that fibre rotation was strongly correlated with the position of the minimum for ST depression (case 2) and ST elevation and that increasing fibre rotation resulted in an anticlockwise rotation of the minimum. Since we obtained these results for a fixed value of ischaemia (30% in the former case and 60% in the latter), we wished to check whether changing the degree of ischaemia has any effect on the position of the minimum.

**Fig. 10:**
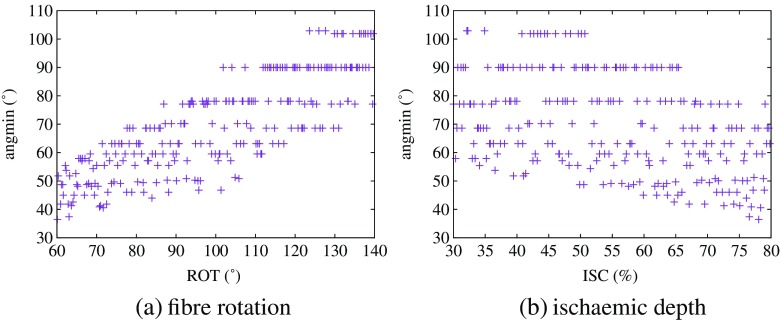
Design data for EPDs of type ST depression (type 2) and ST elevation showing the relationship between the angle of the minimum (angmin) and **a** fibre rotation angle and **b** depth of ischaemia

To ensure that we had EPDs only of either ST depression (type 2) or ST elevation, we set the conductivity values to their means (Table 2) and varied fibre rotation over its usual range (60^∘^ to 140^∘^) and ischaemia from 30 to 80% only. We produced 250 sets of LHC-generated design data and the EPDs corresponding to these and checked that they were all either of type ST depression (type 2) or ST elevation, which they were. Plots of the angle of the minimum against fibre rotation and ischaemic depth are given in Fig. 10a, b, respectively, and these show that not only is there an anticlockwise rotation of the minimum due to fibre rotation, there is also a clockwise rotation due to increasing ischaemic depth. We assessed the relative strengths of these relationships using PLS and found correlations with angmin of 0.89 for fibre rotation and −0.55 for ischaemic depth, indicating that fibre rotation is the stronger effect.

## Discussion

Our modelling study has demonstrated that increasing fibre rotation results, for any one of the three simulated EPD patterns considered, in an anticlockwise rotation of the EPD and, hence, in the position of the outside minima (in the cases where they exist). In addition, we showed that for ST depression (type 2) and ST elevation, the effect of increasing ischaemic depth is clockwise rotation of the minimum. When we assessed the relative effects of the two, fibre rotation was found to have an effect that is approximately 1.5 times stronger than the ischaemic depth.

The effect of ischaemic depth on the angle of the EPD has been reported in an earlier simulation study by Stinstra et al. [55] and the rotation of the EPD with fibre rotation has been also been demonstrated previously [4, 21]. However, in each case, this was only for a particular scenario, not more generally. We also found that none of the conductivity parameters has a significant effect on the position of the minima. These results are significant in terms of the localisation of ST depression, since previous studies have found conflicting results, as discussed in Section 1.

Turning to the effect of conductivities, we found (Table 6) that the blood conductivity *g*_*b*_ has very little effect on the outputs considered. On the other hand, the normal conductivities *g*_*in*_ and *g*_*en*_ have the most significant effect on the potential over the central region for all three types of EPDs and on the magnitude of the outside minimum in types 1 and 2 of ST depression. Also, *g*_*il*_ and *g*_*el*_ are significant in a few cases (Table 6), particularly in relation to the strength of the outside minimum in the case of ST elevation. This is consistent with the results from Table 7 that show the importance of the *g*_*in*_/*g*_*en*_ ratio to the magnitude of the depression in the type 1 and 2 cases and the *g*_*il*_/*g*_*el*_ ratio in the case of ST elevation.

An earlier modelling study [22] pointed out the significance of the *g*_*il*_/*g*_*el*_ and *g*_*it*_/*g*_*et*_ ratios, particularly in relation to the magnitude of the net epicardial potential difference. Since that study used only four bidomain conductivities, the authors could not distinguish between the transverse and normal conductivity ratios. If we assume that *g*_*it*_/*g*_*et*_ is *g*_*in*_/*g*_*en*_, then our results generally agree with the study by Hopenfeld et al. [22], who concluded that an increase in *g*_*il*_/*g*_*el*_ results in an increase in epicardial potential magnitudes for all degrees of ischaemia. This is consistent with our results (Table 6) for ominV and cmaxV for ST depression (type 2) and ST elevation (deepening of the outside minima and increasing the central maximum), but not for ST depression (type 1) where *g*_*in*_/*g*_*en*_ is the only ratio that affects cminV (and results in an increase of its magnitude).

Previous simulation studies [22, 43] have also highlighted the importance of the anisotropy ratios *g*_*il*_/*g*_*it*_ and *g*_*el*_/*g*_*et*_ in ST depression. Potse et al. [43] have suggested that both a decreased ratio for *g*_*il*_/*g*_*it*_ and an increased ratio for *g*_*el*_/*g*_*et*_ can result in ST depression. However, Hopenfeld et al. [22] suggest that different behaviours occurs as a result of changes in these ratios, depending on the ischaemic depth [22]. That is, at 10%, ischaemia increases in *g*_*el*_/*g*_*et*_ result in increased epicardial potential magnitudes but at higher ischaemia, the opposite occurs, and, conversely, at 10%, ischaemia increases in *g*_*il*_/*g*_*it*_ result in decreased epicardial potential magnitudes with increases at higher ischaemia. The reason for this is the paths the injury currents take in the various scenarios [5, 22].

These results do not simply translate to ours if *t* is replaced by *n* (Table 7) or if we leave *t* in the ratios. For example, if we regard the ST depression (type 1) scenario as being roughly equivalent to the 10% ischaemia case, then for *g*_*il*_/*g*_*it*_ and *g*_*il*_/*g*_*in*_, the sign of the correlation is the same for cmaxV and ominV, which means that we cannot have the magnitude of both increasing. This is also true for *g*_*il*_/*g*_*in*_ for ST depression (type 2) and ST elevation. However, we can say that ST depression deepens (the magnitudes, of cminV for (type 1) and ominV for (type 2) and ST elevation, increase) with an increase in *g*_*il*_/*g*_*it*_ and decreases with an increase of *g*_*il*_/*g*_*in*_ in both cases of ST depression and with *g*_*el*_/*g*_*en*_ for the ST elevation case.

We also note that the previous experimental study and modelling study of Li et al. [37] and some modelling studies [21, 39] found that ST depression is located over the lateral boundary between the ischaemic and normal tissue. This is clearly the case for the ST depression (type 2) and ST elevation scenarios (see Section 3.1). However, in the case of ST depression (type 1), a single minimum is found over the ischaemic region, not the lateral boundary. This scenario has previously been identified [22] as occurring for very small ischaemic depths (< 20). We have shown that ST depression (type 1) can occur for a much wider range of ischaemic depths, depending on the conductivity values (particularly for high *g*_*in*_ and low *g*_*en*_ and *g*_*il*_). If ischaemic changes to conductivity values are such that this can occur, then this may explain, for a wide range of ischaemic depths, why it is not always the case that ST depression is located over the boundaries of the ischaemic region.

However, when drawing conclusions from this work, it is worth bearing in mind that we have used a simplified model geometry and made certain choices for the form of the transmembrane potential and ischaemic border zones. For example, the choice of a sharp ischaemic border zone (approximately 1 mm, compared with experimental results that suggest values of around 8–10 mm [14, 31]) is a possible limitation of this work, as is the model setup that assumes that the sheets of fibres start and then remain parallel with the epicardium throughout the ventricular wall [36].

What have presented here is a proof-of-concept study that demonstrates the use of various uncertainty and sensitivity analysis methods in a model of cardiac ischaemia. The approaches used in this study could now be extended to more detailed models of myocardial ischaemia that incorporate a realistic heart and torso anatomy, and these studies may then produce results that have clinical relevance.

As a final comment, we note that the results in this study were found using a combination of complementary analysis methods. Here, we used generalised polynomial chaos in a more qualitative fashion than the other two techniques (GP emulators and PLS), since it is ideally suited to producing pictures of ‘average’ EPDs. Although it is possible to produce quantitative measures of sensitivity for generalised polynomial chaos (Sobol indices [15]), it is somewhat problematic to do so when it is necessary to use subsets of the data, as in this work.

This is not an issue for either GP emulators or PLS, as we demonstrated here. We found that, in almost every case, the two techniques were in agreement about which input variables had a significant effect on an output, although the strength of the effect could not be directly compared. One advantage of PLS over GP emulators is that the direction of the effect is given as part of the calculation, whereas the GP sensitivity indices are unsigned.

## Conclusion

In this work, we used a number of methods (generalised polynomial chaos, Gaussian Process emulators and partial least squares regression) to study and quantify the effect of uncertainty in eight input parameters on outputs, such as the position and magnitude of the maxima and minima, when studying subendocardial ischaemia in a rectangular slab of ventricular tissue using a bidomain model. We found that the three ‘typical’ EPD patterns (a single minimum located over the ischaemic (central) region; a tripole of three minima, with the central minimum now weaker than those outside the central region; and a central maximum, flanked by two minima along the lateral boundaries) could occur for a much wider range of subendocardial ischaemic depths than previously thought, depending on the conductivity values.

Our results showed that the only parameters that affect the magnitude of ST depression are the conductivities *g*_*il*_,*g*_*el*_,*g*_*in*_ and *g*_*en*_ and their ratios, *g*_*il*_/*g*_*el*_ and *g*_*in*_/*g*_*en*_, and not *g*_*it*_/*g*_*et*_.

We found that ST depression deepens for increases in the ratios *g*_*il*_/*g*_*el*_ and *g*_*in*_/*g*_*en*_, where *g*_*in*_/*g*_*en*_ is the only significant one for ST depression (type 1), the two have a similar effects for ST depression (type 2) and *g*_*il*_/*g*_*el*_ is more significant for ST elevation. We also found that the position of the minimum is strongly correlated (anticlockwise) with the value of fibre rotation and less strongly with the ischaemic depth (clockwise), but not with the conductivity values.

Possible future work could include comparing these results with those produced with a more realistic heart model and perhaps varying the conductivities in the ischaemic region over a different range than in the rest of the tissue.

## Electronic supplementary material

Below is the link to the electronic supplementary material.

(PDF 527 KB)

## References

[1] Abdi H (2003) Partial least squares regression (PLS-regression), pp 792–795. Encyclopedia for research methods for the social sciences. Sage

[2] Arthur RM, Geselowitz DB (1970). Effect of inhomogeneities on the apparent location and magnitude of a cardiac current dipole source. IEEE Trans Biomed Eng.

[3] Ayache N, Delingette H, Sermesant M (eds) (2009) A quantitative comparison of the myocardial fibre orientation in the rabbit as determined by histology and by diffusion tensor-MRI. Functional imaging and modeling of the heart: 5th international conference

[4] Barnes JP (2013) Mathematically modeling the electrophysiological effects of ischaemia in the heart. Ph.D. thesis, Griffith University, Brisbane, Australia

[5] Barnes JP, Johnston PR (2010) The effect of the shape of ischaemic regions in the heart on the resulting extracellular epicardial potential distributions. In: Murray A (ed) Computing in cardiology, vol 37. IEEE, IEEE Press, pp 177–180

[6] Barnes JP, Johnston PR (2012). The effect of ischaemic region shape on epicardial potential distributions in transient models of cardiac tissue. ANZIAM J.

[7] Bastos LS, O’Hagan A (2009). Disgnostics for gaussian process emulators. Technometrics.

[8] Bauer S, Edelmann JC, Seemann G, Sachse FB, Dössel O (2013) Estimating intracellular conductivity tensors from confocal microscopy of rabbit ventricular tissue. Biomedizinische Technik/Biomedical Engineering 58 10.1515/bmt-2013-4333 24043045

[9] Becker W, Oakley JE, Surace C, Gili P, Rowson J, Worden K (2012). Bayesian sensitvity analysis of a nonlinear finite element model. Mech Syst Signal Process.

[10] Burton BM, Tate JD, Good W, MacLeod RS (2016) The role of reduced left ventricular, systolic blood volumes in ST segment potentials overlying diseased tissue of the ischaemic heart. In: Murray A (ed) Computing in cardiology, vol 43, pp 209–212 PMC5404699 28451591

[11] Caldwell BJ, Trew ML, Sands GB, Hooks DA, LeGrice IJ, Smaill BH (2009). Three distinct directions of intramural activation reveal nonuniform side–to–side electrical coupling of ventricular myocytes. Circ Arrhythm Electrophysiol.

[12] Chang ETY, Strong M, Clayton RH (2015). Bayesian sensitivity analysis of a cardiac cell model using a Gaussian Process emulator. PLoS ONE.

[13] Clerc L (1976). Directional differences of impulse spread in trabecular muscle from mammalian heart. J Physiol.

[14] Coronel R, Wilms-Schopman FJG, Opthof T, van Capelle FJL, Janse MJ (1991). Injury current and gradients of diastolic stimulation threshold, TQ potentail, and extracellular potassium concentration during acute regional ischemia in the isolated perfused pig heart. Circ Res.

[15] Crestaux T, Le Maitre O, Martinez JM (2009). Polynomial chaos expansion for sensitivity analysis. Reliab Eng Syst Saf.

[16] Geladi P, Kowalski BR (1986). Partial least squares regression: a tutorial. Anal Chim Acta.

[17] Geneser SE, Kirby RM, MacLeod RS (2008). Application of stochastic finite element methods to study the sensitivity of ECG forward modeling to organ conductivity. IEEE Trans Biomed Eng.

[18] Hand PE, Griffith BE, Peskin CS (2009). Deriving macroscopic myocardial conductivities by homogenization of microscopic models. Bull Math Biol.

[19] Hooks D (2007). Myocardial segment-specific model generation for simulating the electrical action of the heart. BioMedical Engineering OnLine.

[20] Hooks DA, Trew ML, Caldwell BJ, Sands GB, LeGrice IJ, Smaill BH (2007). Laminar arrangement of ventricular myocytes influences electrical behavior of the heart. Circ Res.

[21] Hopenfeld B, Stinstra JG, MacLeod RS (2004). Mechanism for ST depression associated with contiguous subendocardial ischaemia. J Cardiovasc Electrophysiol.

[22] Hopenfeld B, Stinstra JG, MacLeod RS (2005). The effect of conductivity on ST-segment epicardial potentials arising from subendocardial ischemia. Ann Biomed Eng.

[23] Johnston BM (2016). Six conductivity values to use in the bidomain model of cardiac tissue. IEEE Trans Biomed Eng.

[24] Johnston BM, Barnes JP, Johnston PR (2016) The effect of conductivity values on activation times and defibrillation thresholds. In: Murray A (ed) Computing in cardiology, vol 43, pp 161–164

[25] Johnston PR (2010). A finite volume method solution for the bidomain equations and their application to modelling cardiac ischaemia. Comput Methods Biomech Biomed Engin.

[26] Johnston PR (2014) Defibrillation thresholds: a generalised polynomial chaos study. In: Murray A (ed) Computing in cardiology, vol 41. IEEE, IEEE Press, pp 197–200

[27] Johnston PR, Kilpatrick D (2003). The effect of conductivity values on ST segment shift in subendocardial ischaemia. IEEE Trans Biomed Eng.

[28] Johnston PR, Kilpatrick D, Li CY (2001). The importance of anisotropy in modelling ST segment shift in subendocardial ischaemia. IEEE Trans Biomed Eng.

[29] Johnstone RH, Chang ETY, Bardenet R, de Boer TP, Gavaghan DJ, Pathmanathan P, Clayton RH, Mirams GR (2016). Uncertainty and variability in models of the cardiac action potential: can we build trustworthy models?. J Mol Cell Cardiol.

[30] Kilpatrick D, Johnston PR, Li DS (2003). Mechanisms of ST change in partial thickness ischemia. J Electrocardiol.

[31] Kléber AG, Janse MJ, van Capelle FJL, Durrer D (1978). Mechanism and time course of s-T and t–Q segment changes during acute regional myocardial ischemia in the pig heart determined by extracellular and intracellular recordings. Circ Res.

[32] Kontos MC, Diercks DB, Kirk JD (2010). Emergency department and office-based evaluation of patients with chest pain. Mayo Clin Proc.

[33] Krassowska W, Neu JC (1992) Theoretical versus experimental estimates of the effective conductivities of cardiac muscle. In: Computers in cardiology 1992. IEEE, pp 703–706

[34] Le Guyader P, Savard P, Trelles F (1997) Measurement of myocardial conductivities with a four–electrode technique in the frequency domain. In: Proceedings of 19th international conference. IEEE/EMBS, pp 2448–2449

[35] Le Guyader P, Trelles F, Savard P (2001). Extracellular measurement of anisotropic bidomain myocardial conductivities. I. Theoretical analysis. Ann Biomed Eng.

[36] LeGrice IJ, Hunter PJ, Smail BH (1997). Laminar structure of the heart: a mathematical model. Am J Physiol.

[37] Li D, Li CY, Yong AC, Kilpatrick D (1998). Source of electrocardiographic ST changes in subendocardial ischemia. Circ Res.

[38] Lombaert H, Peyrat J, Croisille P, Rapacchi S, Fanton L, Cheriet F, Clarysse P, Magnin I, Delingette H, Ayache N (2012). Human atlas of the cardiac fiber architecture: study on a healthy population. IEEE Trans Med Imaging.

[39] MacLachlan MC, Sundnes J, Lines GT (2005). Simulation of ST segment changes during subendocardial ischemia using a realistic 3-D cardiac geometry. IEEE Trans Biomed Eng.

[40] Macleod RS, Shome S, Stinstra J, Punske BB, Hopenfeld B (2005). Mechanisms of ischemia-induced ST-segment changes. J Electrocardiol.

[41] Muszkiewicz A, Britton OJ, Gemmell P, Passini E, Sánchez C, Zhou X, Carusi A, Quinn TA, Burrage K, Bueno-Orovio A, Rodrigueza B (2016). Variability in cardiac electrophysiology: using experimentally-calibrated populations of models to move beyond the single virtual physiological human paradigm. Prog Biophys Mol Biol.

[42] Oakley JE, O’Hagan A (2004). Probabilistic sensitivity analysis of complex models: a Bayesian approach. J R Stat Soc Ser B (Stat Methodol).

[43] Potse M, Coronel R, Falcao S, LeBlanc AR, Vinet A (2007). The effect of lesion size and tissue remodeling on ST deviation in partial-thickness ischemia. Heart Rhythm.

[44] Potse M, Coronel R, LeBlanc AR, Vinet A (2007). The role of extracellular potassium transport in computer models of the ischemic zone. Med Biol Eng Comput.

[45] Roberts DE, Hersh LT, Scher AM (1979). Influence of cardiac fiber orientation on wavefront voltage, conduction velocity and tissue resistivity in the dog. Circ Res.

[46] Roberts DE, Scher AM (1982). Effects of tissue anisotropy on extracellular potential fields in canine myocardium in situ. Circ Res.

[47] Roth BJ (1988). The electrical potential produced by a strand of cardiac muscle: a bidomain analysis. Ann Biomed Eng.

[48] Roth BJ (1997). Electrical conductivity values used with the bidomain model of cardiac tissue. IEEE Trans Biomed Eng.

[49] Roth BJ (1997). Nonsustained reentry following successive stimulation of cardiac tissue through a unipolar electrode. J Cardiovasc Electrophysiol.

[50] Roth BJ (2000). An s1 gradient of refractoriness is not essential for reentry induction by an s2 stimulus. IEEE Trans Biomed Eng.

[51] Rush S, Abildskov JA, McFee R (1963). Resistivity of body tissues at low frequencies. Circ Res.

[52] Smolyak SA (1963). Quadrature and interpolation formulas for tensor products of certain classes of function. Soviet Mathematics Doklady.

[53] Sobie E (2009). Parameter sensitivity analysis in electrophysiological models using multivariate regression. Biophys J.

[54] Stinstra J, Hopenfeld B, MacLeod R (2005). On the passive cardiac conductivity. Ann Biomed Eng.

[55] Stinstra JG, Hopenfeld B, Macleod RS (2004) Using models of the passive cardiac conductivity and full heart anisotropic bidomain to study the epdicardial potentials in ischaemia. In: Proceedings of the IEEE engineering in medicine and biology 26th annual international conference. IEEE EMBS, IEEE Press, pp 3555–3558 10.1109/IEMBS.2004.1403999 17271058

[56] Stinstra JG, Shome S, Hopenfeld B, MacLeod RS (2005). Modelling passive cardiac conductivity during ischaemia. Med Biol Eng Comput.

[57] Streeter D, Spotnitz H, Patel D, Ross J, Sonnenblick E (1969). Fiber orientation in the canine left ventricle during diastole and systole. Circ Res.

[58] Swenson D, Geneser S, Stinstra J, Kirby R, MacLeod R (2011). Cardiac position sensitivity study in the electrocardiographic forward problem using stochastic collocation and boundary element methods. Ann Biomed Eng.

[59] Taccardi B, Macchi E, Lux RL, Ershler PR, Spaggiari S, Baruffi S, Vyhmeister Y (1994). Effect of myocardial fiber direction on epicardial potentials. Circulation.

[60] Trayanova N, Eason J, Aguel F (2002). Computer simulations of cardiac defibrillation: a look inside the heart. Comput Vis Sci.

[61] Tung L (1978) A bi-domain model for describing ischaemic myocardial D-C potentials. Ph.D. thesis, Massachusetts Institute of Technology

[62] Xiu D (2007). Efficient collocational approach for parametric uncertainty analysis. Commun Comput Phys.

[63] Xiu D, Hesthaven JS (2005). High-order collocation methods for differential equations with random inputs. SIAM J Sci Comput.

