# Supplementary material for: Quantifying the effect of uncertainty in input parameters in a simplified bidomain model of partial thickness ischaemia
Source: Med Biol Eng Comput. 2017 Sep 20;56(5):761–80. doi: 10.1007/s11517-017-1714-y (PMC5906519; doi:10.1007/s11517-017-1714-y)
Supplement: Supplementary file 1 — (PDF 527 KB) [file 11517_2017_1714_MOESM1_ESM.pdf]

Noname manuscript No.
(will be inserted by the editor)

Quantifying the effect of uncertainty in input parameters in a simplified bidomain model of partial thickness ischaemia – Supplementary data

Barbara M. Johnston · Sam Coveney · Eugene T.Y. Chang · Peter R. Johnston · Richard H. Clayton

Received: date / Accepted: date

B. Johnston
Queensland Micro– and Nanotechnology Centre and School of Natural Sciences,
Griffith University, Nathan, QLD, 4111, Australia
Tel.: +61-7-37354405
Fax: +61-7-37357656
E-mail: : Barbara.Johnston@griffith.edu.au.

S. Coveney
Department of Physics and Astronomy,
University of Sheffield, Sheffield, United Kingdom

E. Chang
Department of Computer Science and INSIGNEO Institute for *in–silico* Medicine,
University of Sheffield, Sheffield, United Kingdom

P. Johnston
Queensland Micro– and Nanotechnology Centre and School of Natural Sciences,
Griffith University, Nathan, QLD, 4111, Australia

R. Clayton
Department of Computer Science and INSIGNEO Institute for *in–silico* Medicine,
University of Sheffield, Sheffield, United Kingdom

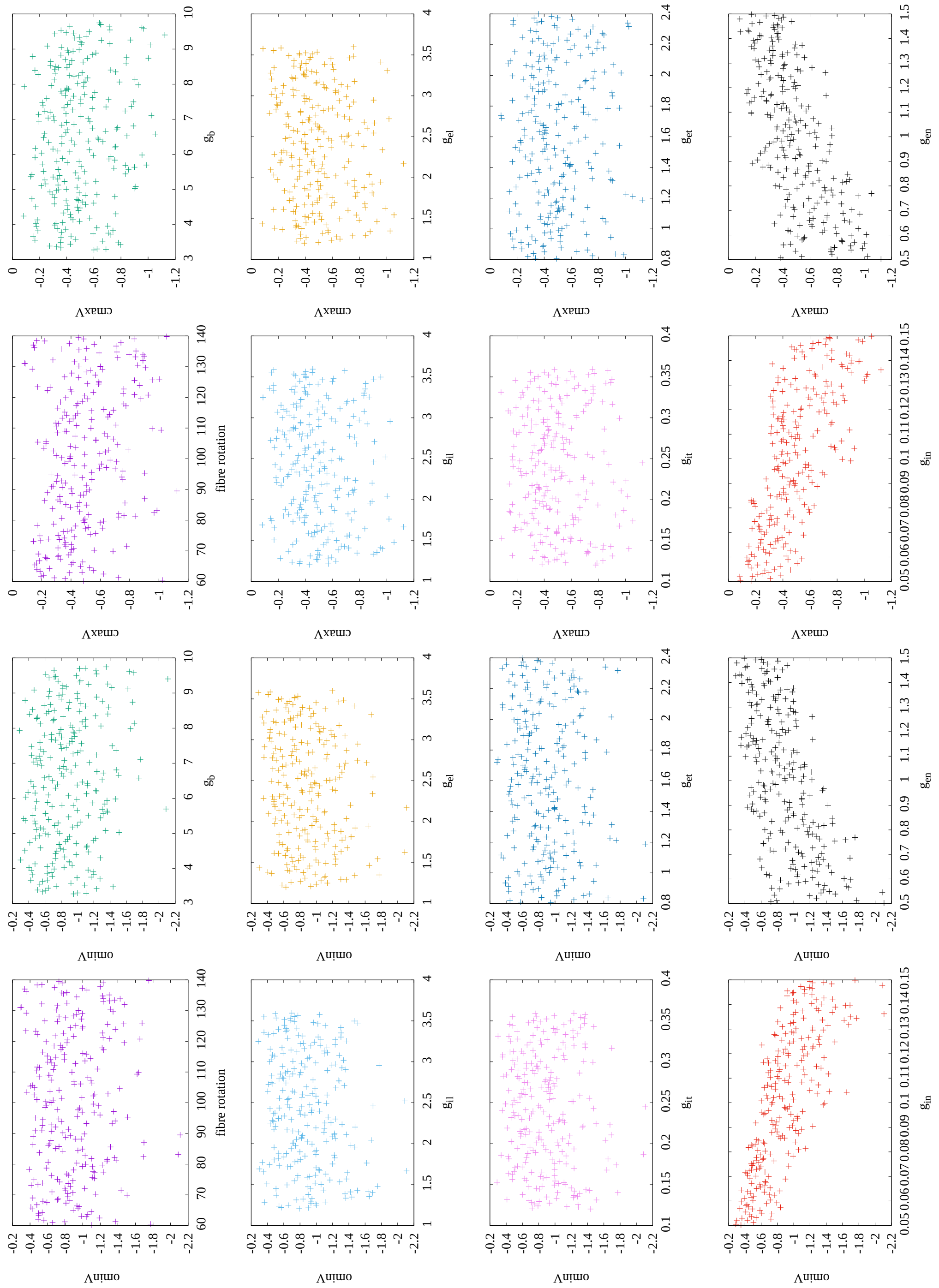

Fig. 1 Design data for the features, outside centre minimum voltage (ominV) and centre maximum voltage (cmaxV), in mV, of the epicardial potential distributions of ST depression (type 1). These are plotted against each of the eight input variables, with units of mS/cm for conductivities and degrees for fibre rotation.

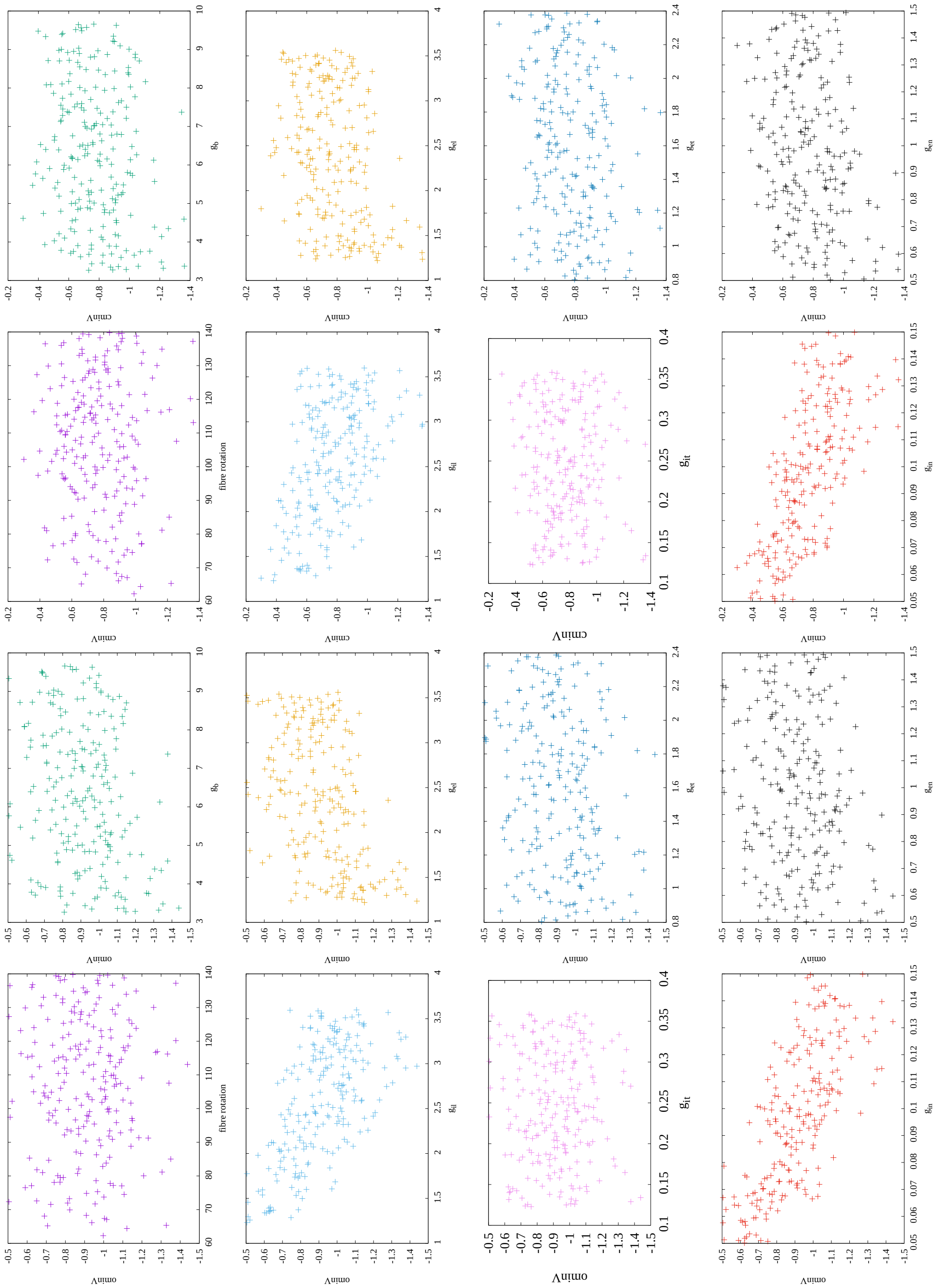

Fig. 2 Design data for the features outside centre minimum voltage (ominV) and centre minimum voltage (cminV), in mV, of the epicardial potential distributions of ST depression (type 2). These are plotted against each of the eight input variables, with units of mS/cm for conductivities and degrees for fibre rotation.

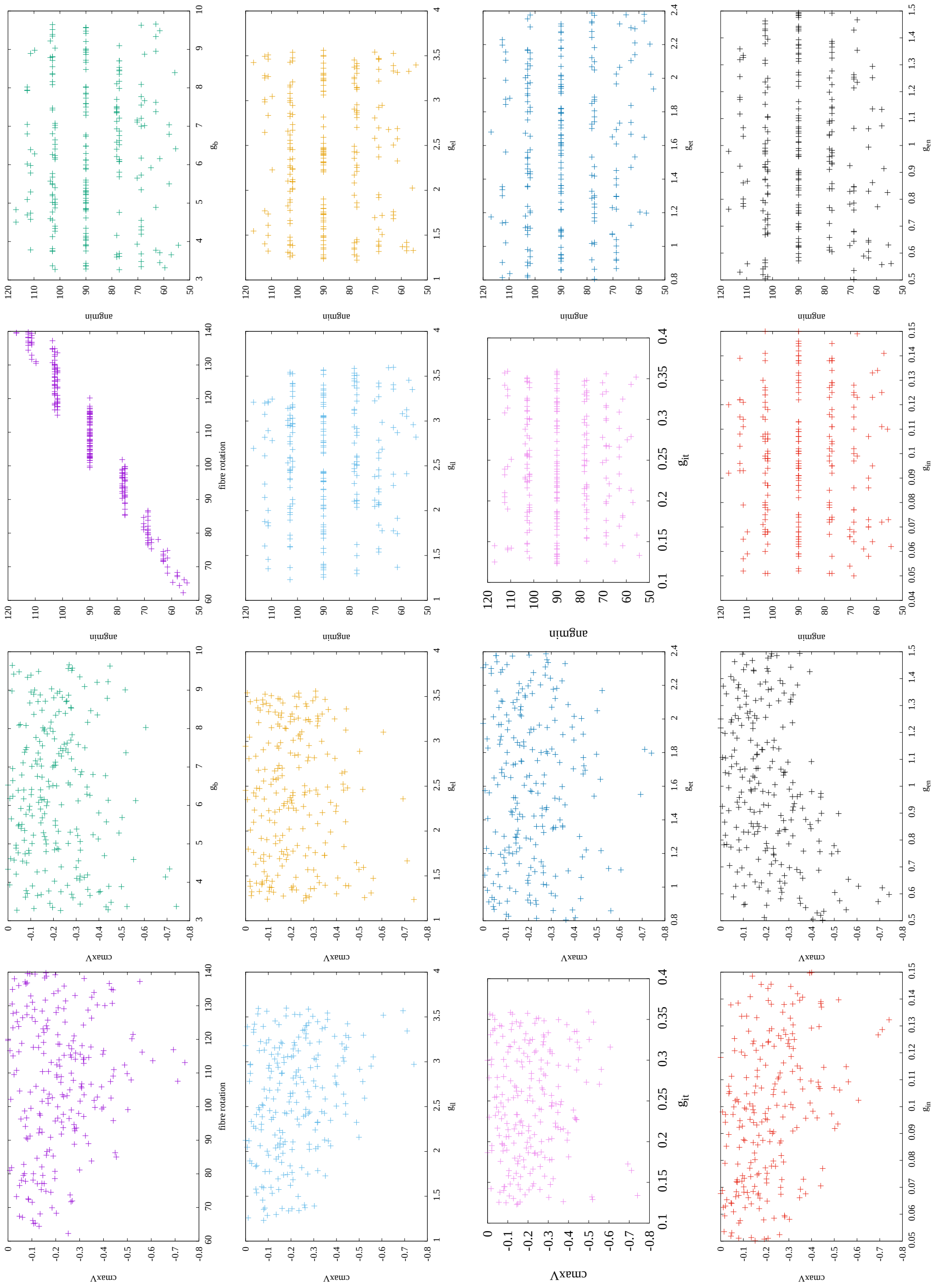

Fig. 3 Design data for the features centre maximum voltage (cmaxV), in mV, and angle of the minimum with the x–axis (angmin), in degrees, of the epicardial potential distributions of ST depression (type 2). These are plotted against each of the eight input variables, with units of mS/cm for conductivities and degrees for fibre rotation.

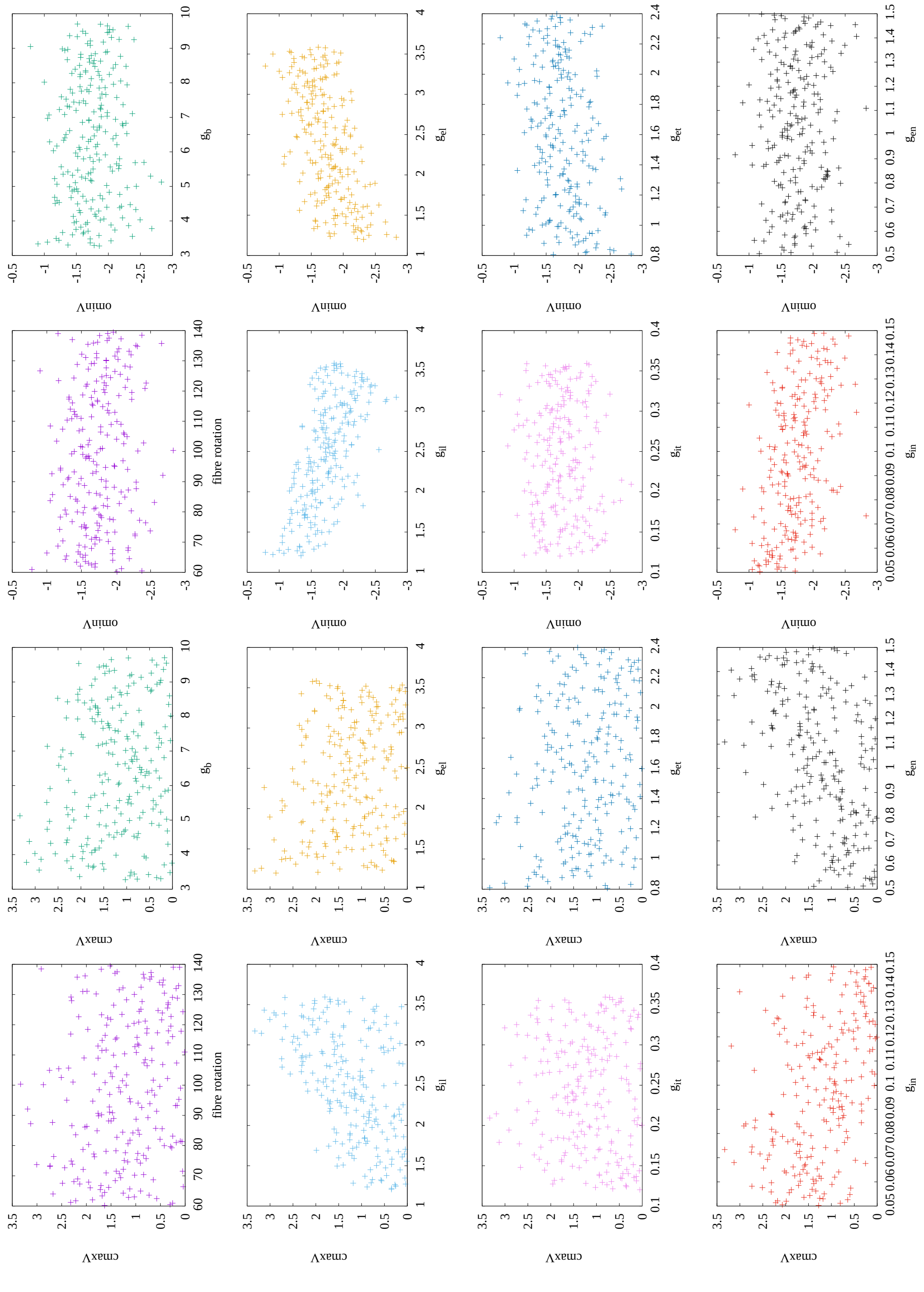

Fig. 4 Design data for the features centre maximum voltage (cmaxV) and outside centre minimum voltage (ominV), in mV, of the ST elevation type epicardial potential distributions. These are plotted against each of the eight input variables, with units of mS/cm for conductivities and degrees for fibre rotation.

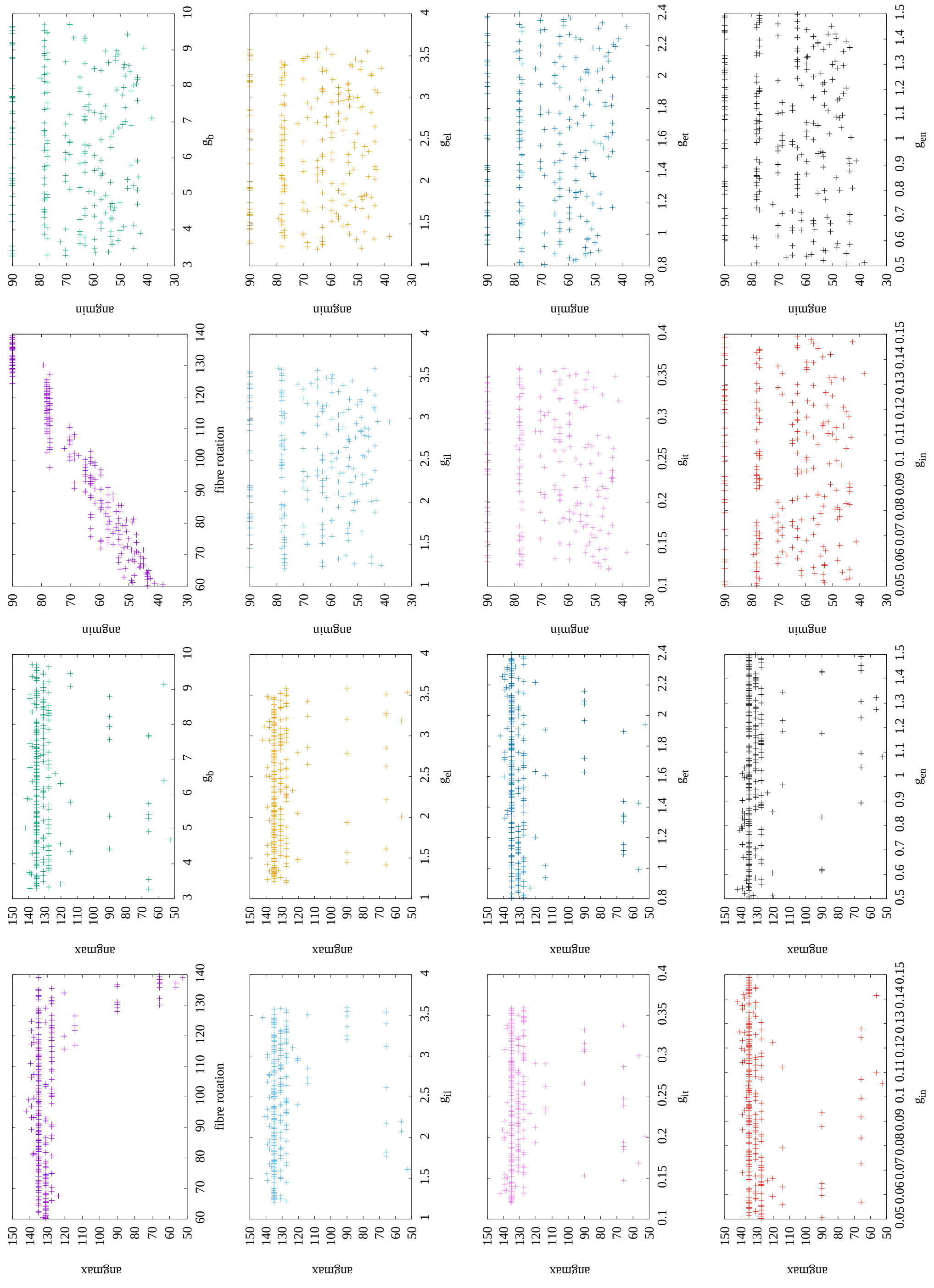

Fig. 5 Design data for the features angle of the maximum (angmax) and angle of the minimum with the x–axis (angmin), in degrees, of the ST elevation type epicardial potential distributions. These are plotted against each of the eight input variables, with units of mS/cm for conductivities and degrees for fibre rotation.
